# Structural, Thermal, and Vibrational Properties of N,N-Dimethylglycine–Chloranilic Acid—A New Co-Crystal Based on an Aliphatic Amino Acid

**DOI:** 10.3390/ma14123292

**Published:** 2021-06-14

**Authors:** Joanna Hetmańczyk, Łukasz Hetmańczyk, Joanna Nowicka-Scheibe, Andrzej Pawlukojć, Jan K. Maurin, Wojciech Schilf

**Affiliations:** 1 Faculty of Chemistry, Jagiellonian University, Gronostajowa 2 Str., 30-387 Cracow, Poland; lukasz.hetmanczyk@uj.edu.pl; 2 Department of Organic and Physical Chemistry, Faculty of Chemical Technology and Engineering, West Pomeranian University of Technology, Piastów Ave. 42, 71-065 Szczecin, Poland; joanna.nowicka-scheibe@zut.edu.pl; 3 Institute of Nuclear Chemistry and Technology, Dorodna 16 Str., 03-195 Warsaw, Poland; andrzej@jinr.ru; 4 Frank Laboratory of Neutron Physics, Joint Institute for Nuclear Research, 141-980 Dubna, Russia; 5 National Medicines Institute, Chełmska 30/34 Str., 00-725 Warsaw, Poland; j.maurin@nil.gov.pl; 6 National Centre for Nuclear Research, Sołtana 7 Str., 05-400 Otwock, Poland; 7 Institute of Organic Chemistry Polish Academy of Sciences, Kasprzaka 44, 01-224 Warsaw, Poland; wojciech.schilf@icho.edu.pl

**Keywords:** N,N-dimethylglycine–chloranilic acid (DMG^+^–CLA^−^) co-crystal, X-ray diffraction, inelastic neutron scattering (INS), temperature dependent IR spectroscopy, DFT calculation

## Abstract

The new complex of N,N-Dimethylglycine (DMG) with chloranilic acid (CLA) was synthesized and examined for thermal, structural, and dynamical properties. The structure of the reaction product between DMG and CLA was investigated in a deuterated dimethyl sulfoxide (DMSO-d6) solution and in the solid state by Nuclear Magnetic Resonance (NMR) (Cross Polarization Magic Angle Spinning-CPMAS NMR). The formation of the 1:1 complex of CLA and DMG in the DMSO solution was also confirmed by diffusion measurement. X-ray single crystal diffraction results revealed that the N,N-dimethylglycine–chloranilic acid (DMG^+^–CLA^−^) complex crystallizes in the centrosymmetric triclinic P-1 space group. The X-ray diffraction and NMR spectroscopy show the presence of the protonated form of N,N-dimethylglycine and the deprotonated form of chloranilic acid molecules. The vibrational properties of the co-crystal were investigated by the use of neutron (INS), infrared (IR), and Raman (RS) spectroscopies, as well as the density functional theory (DFT) with periodic boundary conditions. From the band shape analysis of the N–CH_3_ bending vibration, we can conclude that the CH_3_ groups perform fast (τ_R_ ≈ 10^−11^ to 10^‒13^ s) reorientational motions down to a temperature of 140 K, with activation energy at ca. 6.7 kJ mol^−1^. X-ray diffraction and IR investigations confirm the presence of a strong N^+^–H···O^−^ hydrogen bond in the studied co-crystal.

## 1. Introduction

For almost 50 years N,N-Dimethylglycine (DMG) has been known as a widely used nutritional supplement in health fields, especially as a key ingredient of vitamin B-15 and as a product of the metabolism pathway of choline and methionine [1,2,3]. DMG, according to a lot of research, could positively influence the immune response in laboratory animals and humans [4], boost physical and mental performance, and enhance cardiovascular function in clinical patients. For this reason, DMG is a well-known element of supplements and medicaments and is recognized for protecting the liver by aiding in detoxification. Previously completed works are increasing our understanding of the prophylactic and therapeutic uses of DMG as a metabolic enhancer and immunomodulator. N,N-Dimethylglycine is a tertiary amino acid, the dimethylated derivative of glycine (Gly) forms molecular salts and adducts with various inorganic and organic acids [5,6,7,8], which reveal interesting physical properties leading to ferroelectric, antiferroelectric, and ferroelastic phases [9]. For several years, the salts of dimethylglycine with various inorganic and organic acids have been objects of intensive analysis [9,10,11]. Clarke et al. synthesized Dimethylglycine and its first salt dimethylglycine hydrochloride in 1933 [12]. Rodrigues et al. obtained the trifluoroacetate of dimethylglycine [13]. Anioła et al. studied the reaction of glycine, sarcosine, dimethylglycine, and betaine with squaric acid [2]. Hence, we aimed to obtain an N,N-Dimethylglycine complex with chloranilic acid (CLA). Since amino acids and their complexes are becoming increasingly popular, we proceeded with long-term research on chloranilic acid complexes with several amino acids, such as L-leucine and D-leucine [14,15]. Chloranilic acid as a significant proton donor shows simultaneously marked electron affinity as a quinoid system. CLA forms supramolecular phenomena possessing two equivalent OH groups [16,17]. The proton transfer process is still not well-understood despite the fact that it is considered as one of the most common transformations in nature. Chloranilic acid and amino acids form complexes with the proton transfer. Due to their dipolar nature caused by the presence of carboxylate anion COO^−^ and protonated amino NH_3_^+^ groups, as well as their large hyperpolarizability and chiral α carbon atom, most of the amino acids exhibit physical and chemical properties, which make them suitable material for the production of nonlinear optical (NLO) application devices. Additionally, this makes amino acids and their salts objects of diversified and intensive studies.

The vibrational properties of N,N-Dimethylglycine (DMG) were characterized thoroughly by using IR, inelastic neutron scattering (INS), and NMR spectroscopy [18,19,20,21,22]. The dynamical properties of chloranilic acid, which is simultaneously a strong proton donor and an electron acceptor, were analyzed using experimental (IR, Raman, and INS) and theoretical (DFT) methods [23].

From the point of view of materials science, information on the crystal structures of the complex between the N,N-dimethyl derivative of glycine and chloranilic acid is essential for understanding the role of hydrogen bonds and other intermolecular forces in the association of ions in the crystalline state. A new complex is characterized by X-ray diffraction. Inelastic neutron scattering (INS), infrared (IR), Raman (RS), and ^1^H, ^13^C and ^15^N NMR spectroscopy methods were used to determine the dynamic structure of the N,N-Dimethylglycine–chloranilic acid complex (DMG^+^–CLA^−^). The structure of the complexation product of chloranilic acid (CLA) and N,N-dimethylglycine (DMG) was investigated in both a DMSO solution and in the solid state by NMR (CPMAS NMR). Additionally, DFT calculation with periodic boundary conditions for an experimental data analysis was used.

## 2. Materials and Methods

The substrates (N,N-Dimethylglycine and chloranilic acid) were purchased from Merck (formerly Sigma Aldrich, Merck Sp. z o.o., an affiliate of Merck KGaA, Darmstadt, Germany). They were used as delivered, without any purification. The resultant sample was obtained by mixing an equimolar (1:1) ratio of N,N-Dimethylglycine (1.03 g), which was dissolved in double distilled water, with chloranilic acid (2.08 g) dissolved in acetone. The chloranilic acid was added dropwise to DMG. Next, the obtained mixture was stirred well for about 2–3 h at 50 °C until a homogeneous solution was formed. Finally, the solution was filtered and kept undisturbed at ambient temperature. After a few weeks, the crystalline material was collected and recrystallized from a mixture of double distilled water and ethanol (1:4 ratio).

The crystal structure of the N,N-dimethylglycine–chloranilic acid co-crystal was solved from single crystal X-ray diffraction measurements. The color of the sample was dark violet and the crystals were needle-shaped. The data were collected at room temperature using a Xcalibur R diffractometer (Oxford Diffraction, Oxford, UK). The characteristic CuKα monochromatic radiation and Ruby ccd detector (Oxford Diffraction, Oxford, UK) were employed in the data acquisition. The CrysAlisPro software [24] was used for data collection strategy, data reduction, and lattice constant refinement. Structure was solved using the SHELXS-97 software (University of Göttingen, Göttingen, Germany) and refined using the SHELXL-97 program [25]. All H atoms were located using difference Fourier maps and refined freely. Their isotropic displacement parameters were tied to the respective Ueq values for the nitrogen atoms and equal to 1.2 times Ueq.

The nitrogen and carbon solid-state spectra were measured using the Bruker 500 Avance II spectrometer (Bruker BioSpin GmbH, Rheinstetten, Germany) at room temperature. All CPMAS spectra were collected using a 4 mm MAS H/BB Bruker probehead. The typical acquisition parameters for carbon CPMAS spectra were as follows: spectral width 31.25 kHz, acquisition time 20 ms, contact time 2 ms, rotation rate 10 kHz, and relaxation delay 10 s. About 100 scans were enough to obtain a good signal-to-noise ratio. For short contact time (SCT) experiments, the contact time was reduced to 40 µs. For nitrogen-15 CPMAS experiments, the following acquisition parameters were applied: spectral width 25 kHz, acquisition time 20 ms, contact time 4 ms, rotation rate 6 kHz, and relaxation delay was optimized to obtain the best signal-to-noise ratio. For some samples, up to 3000 scans were collected to obtain a sufficient S/N ratio. The glycine sample was used as the chemical shift standard material and the obtained chemical shift values were recalculated to nitromethane and TMS (tetramethylsilane) scales for nitrogen and carbon measurements, respectively. The cross polarization (CP) sequence was performed using a ramped proton pulse and continuous wave heteroatom irradiation. The modulated TPPM-15 decoupling was used during the acquisition period. All solution-state NMR measurements were run on Varian-Agilent 600 VNMRS and Varian-Agilent 500 VNMRS spectrometers (Agilent Technologies, Inc., Santa Clara, CA, United States). For the 1D carbon spectrum, a 500 MHz instrument with Auto XDB (direct configuration) probehead was used. For proton-detected measurements: ^1^H and ^15^N GHMBC spectra, the 600 MHz instrument with Auto XID (indirect configuration) was applied. For both acquisition and spectrum processing, standard Agilent software (Vnmrj 3.1, Agilent Technologies, Inc., Santa Clara, CA, United States) was used.

Thermogravimetric results (TG) were obtained with the use of the Mettler-Toledo 851^e^ instrument (Mettler-Toledo (Schweiz) GmbH, Greifensee, Switzerland). Measurements were carried out in the argon atmosphere (flow rate 60 mL∙min^−1^) in the temperature range of 25–900 °C, with a constant heating rate equal to 10 °C∙min^−1^. Temperature was measured by Pt-Pt/Rh thermocouple, with an accuracy of ±0.5 °C. A sample with a mass of 6.3400 mg was put into the 150 μL open corundum vessel. The gaseous products of the decomposition were analyzed by a quadrupole Balzers ThermoStar mass spectrometer apparatus.

Differential scanning calorimetry (DSC) analysis was performed using a Mettler-Toledo 821^e^ calorimeter. Measurements were performed with the same heating rate as in the case of TG but in the narrower temperature region of 25–600 °C. The examined sample (mass = 18.5730 mg) was enclosed in a partially open (with micro hole) aluminum vessel (40 μg).

The Raman spectrum was recorded on the MultiRAM FT Raman Bruker spectrometer (Bruker Optik GmbH, Ettlingen, Germany), with a 1064 nm laser (150 mW power) as an excitation source. The resolution was set at 2 cm^−1^. A total of 128 scans (50–3500 cm^−1^ wavenumber) were collected.

The IR vibrational spectra were measured using a Fourier transform Bruker Vertex 70v vacuum spectrometer (Bruker Optik GmbH, Ettlingen, Germany). Two spectral ranges were explored: middle infrared (MIR) and far infrared (FIR). The data were collected in the 4000–400 and 600–30 cm^−1^ wavenumber regions, respectively, with a resolution equal to 2 cm^−1^. Resulting absorption spectra were obtained in a transmission mode by measuring and averaging 32 scans. In the case of MIR measurement, the sample was suspended in a Nujol mull and placed between two KBr discs. The sample prepared in this way was protected against the influence of environmental effects such as vacuum or humidity. Moreover, one can expect that some molecular groups (for example, methyl groups) presented in the studied system may reorientate. This motion can be strongly affected or even blocked when the sample is prepared in the form of a pressed pellet under high pressure (10 tons). In the case of FIR measurements, the sample was prepared in the following manner. About a few milligrams of sample was mixed and grinded with Apiezon N greasy. Next, this mixture was put on a polyethylene (PE) disc. Both Apiezon and PE are transparent to radiation within 600–30 cm^−1^ and do not show significant absorption peaks.

Additionally, one spectrum at room temperature was collected as sample in a KBr pellet form. This was done to check the spectral range covered by the Nujol bands. As stated above, pressing the sample may cause some molecular reorientations to be blocked by matrix effects.

Temperature-dependent IR measurements were performed using an Advanced Research System DE-202A cryostat (Advanced Research System, Inc., Macungie, PA, USA). This device was coupled to an ARS-2HW helium compressor, which works in a closed cycle manner. A diode temperature sensor was mounted near the sample. Temperature was controlled with the LakeShore 331S device, with an accuracy equal to 0.1 K. The sample was mounted at room temperature and IR data were registered upon cooling with a step 10 K. After the desired temperature was reached, the system was left for approximately 3 min to stabilize and after this time, the spectrum was collected. KRS-5 and PE windows were used in the cryostat for MIR and FIR data collection, respectively.

The incoherent inelastic/quasielastic neutron scattering spectra were measured using the time-of-flight method on a NERA spectrometer [26] at the high-flux pulsed reactor IBR-2 in Dubna (Russia) at a temperature of 10 K. The spectra were converted from neutrons per channel to *S*(***Q***, ω) scattering function per energy transfer. At the energy transfer between 5 and 1200 cm^−1^, the relative INS resolution was estimated to be ca. 3%. The *S(**Q***, ω) function (scattering law) for INS, as described in [27,28,29], can be expressed in the form of an isotropic harmonic oscillator:

(1) $$S\left(Q,n\omega_{i}\right)\propto \frac{{\left(QU_{i}\right)}^{2n}}{n!}\cdot exp(-{\left(QU_{Tot}\right)}^{2}),$$

where the ω_i_ and *U_i_* denote the frequency and the mean square displacement of the atoms in the i-th mode, respectively. *Q* stands for the momentum transfer at which the data were gathered. *U_Tot_* describes the total root square displacement of all atoms in all modes. Fundamental modes are for *n* = 1, while *n* ≥ 2 is responsible for overtones. This equation is valid for a hydrogenous material measured at low temperatures, and for a spectrometer working with a neutron energy loss.

Vibrational spectra (IR, RS, INS) were computed for the crystalline solid state within the frame of the DFT method [30,31], using a CASTEP software [32]. As a first step in the calculation, the experimental geometry (taken from our X-ray single crystal diffraction measurements at 293 K) was optimized. Lattice parameters were held constant and only atomic positions were relaxed. The BFGS minimizer was applied [33]. Next, using the density functional perturbation theory, the eigenvalues and eigenmodes were computed [34]. The PBE (Perdew–Burke–Ernzerhof) generalized gradient approximation (GGA) function was employed in computations [35]. To account for van der Waals interactions, the dispersion correction methodology introduced by Tkatchenko–Scheffler [36] was used. The calculations were done at Γ point using on-the-the-fly generated norm-conserving pseudopotentials. Electronic states were sampled on a 2 × 1 × 1 Monkhorst–Pack grid [37] with the plane-wave cut-off energy equal to 830 eV. The convergence threshold for SCF iterations was set at 5 × 10^−10^ eV/atom. The optimization parameters were set as follows: total energy convergence tolerance, 5 × 10^−6^ eV/atom; maximum force tolerance, 0.01 eV/Å; and maximum displacement tolerance, 5 × 10^−4^ Å [38]. The INS spectrum was simulated based on the results obtained from DFT calculations. Equation (1) allows one to predict INS features if the eigenvectors and eigenvalues are known, for example, from DFT calculations. The AbINS [39] plugin for MANTID [40] program was used for this purpose.

## 3. Results and Discussion

### 3.1. X-ray Diffraction Studies

Crystal data, data collection, and structure refinement details for the co-crystal of N,N-dimethylglycine–chloranilic acid at 293 K are shown in Table 1. The crystal structure has been deposited at the Cambridge Crystallographic Data Centre (deposition number CCDC 2069957). This data can be obtained free of charge via http://www.ccdc.cam.ac.uk/conts/retrieving.html.

**Figure 1:**
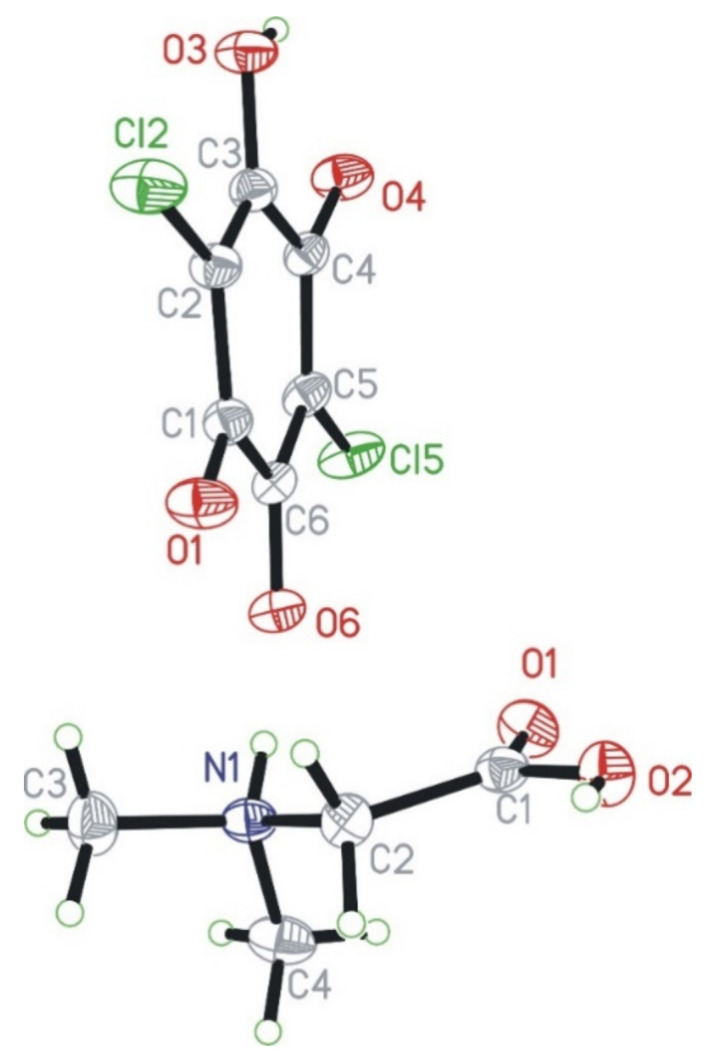
Numbering scheme for N,N-dimethylglycine cation (bottom) and chloranilic acid anion (top). The independent amino acid molecules are denoted with the capital letters A and B, whereas the chloranilic acid are indicated with C and D, respectively. Independent molecules of every kind have very similar geometries.

N,N-dimethylglycine–chloranilic acid co-crystal crystallizes in the centrosymmetric triclinic P-1 space group. The independent part of the unit cell is composed of four ions: two N,N-dimethyl glycine cations (denoted as A and B) and two chloranilic acid anions (denoted as C and D, respectively). The numbering scheme of the molecules is depicted in Figure 1, which shows only one of the cation–anion pairs.

**Figure 2:**
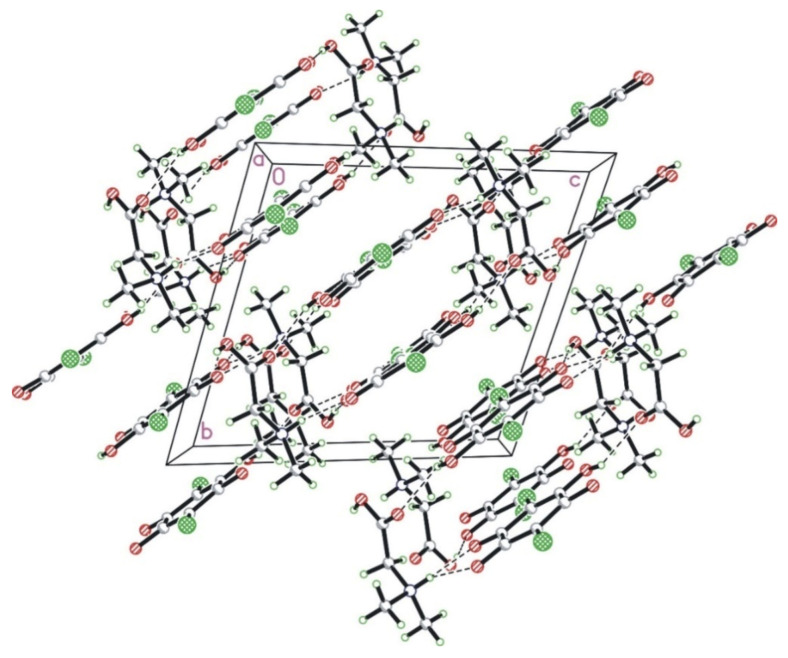
The crystal packing of N,N-dimethylglycine–chloranilic acid 1:1 shown in the *a*-direction. The N–H···O and O–H···O hydrogen bonds are shown as dashed lines.

Cations and anions form a layered structure where the alternating anion and cation layers are perpendicular to the 0, −1, 1 direction. The layers are interlinked with each other in a series of strong N–H···O and O–H···O hydrogen bonds. Interestingly, there are no hydrogen bonds between molecules within a layer (neither chloranilic acid nor amino acid). The crystal packing diagram in the *a*-direction is shown in Figure 2.

The selected bond lengths as well as bond and torsion angles for the co-crystal observed at room temperature and calculated for the optimized geometry (PBE + vdW level) are presented in Table 2. We can see slight differences between the experimental and optimized values (before and after geometry optimization). Generally, the values calculated and determined experimentally for bond length and angles are close to each other. The biggest differences are observed for X–H bond lengths (X:–C, N, O). It is well known that these distances are underestimated in the case of X-ray measurements. The more reliable distances can be obtained from neutron diffraction measurements. Some of the torsion angles are negative. The values of these angles should be in the range of 0–180 degrees. If the value exceeds 180 degrees, then the angle measured in the opposite direction is given. The sign of this angle is associated with the direction in which the angle is given, i.e., if it is clockwise or anticlockwise.

**Figure 3:**
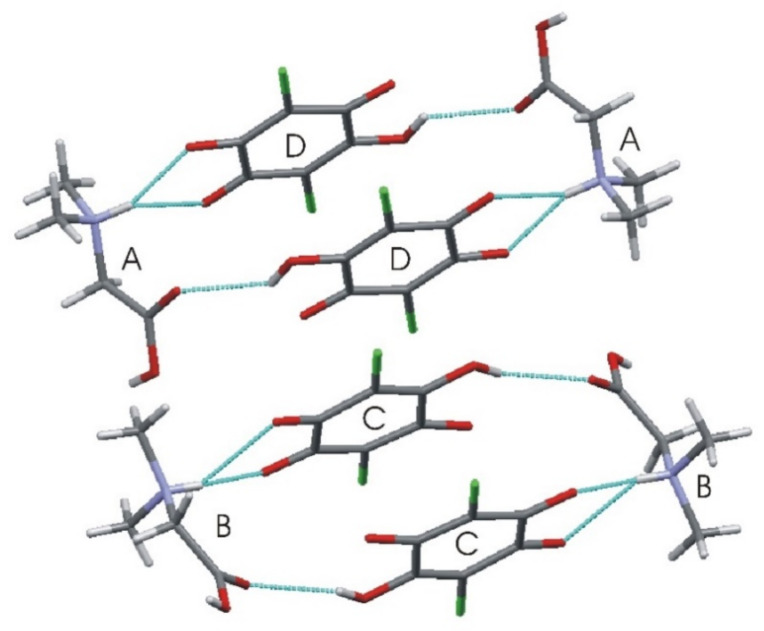
Characteristic ring aggregates in N,N-dimethylglycine–chloranilic acid 1:1.

Table 3 lists the respective hydrogen bond geometry. The N–H amino groups are involved in bifurcated hydrogen bonds, with two neighboring carbonyl oxygen atoms of the chloranilic acid molecule. There are three intermolecular hydrogen bonds for every type of independent molecule: A, B, C, and D. In C and D molecules, there are intramolecular hydrogen bonds: O3D–H3D···O4D and O3–H3C···O4C. However, the same donor in either the C or D molecule also takes part in the intermolecular bonds with molecule A or B, respectively. In this case, the rings D···A···D’···A’ and C···B···C’···B’, or the chains ··D···A···C···B·· are formed; thus, the graphic is described as “chains of rings” (see Figure 3). The structure is also interesting and much more complicated because there are two different intermolecular binary hydrogen bonds with two donors and one acceptor: between molecules A, B (donors) and C or D (acceptor). Therefore, there are also other hydrogen bond chains such as: ··C···B···D···A·· along the crystallographic *a*-direction, where donors belong to the A and B molecules, whereas acceptors are in C and D, respectively.

**Figure 4:**
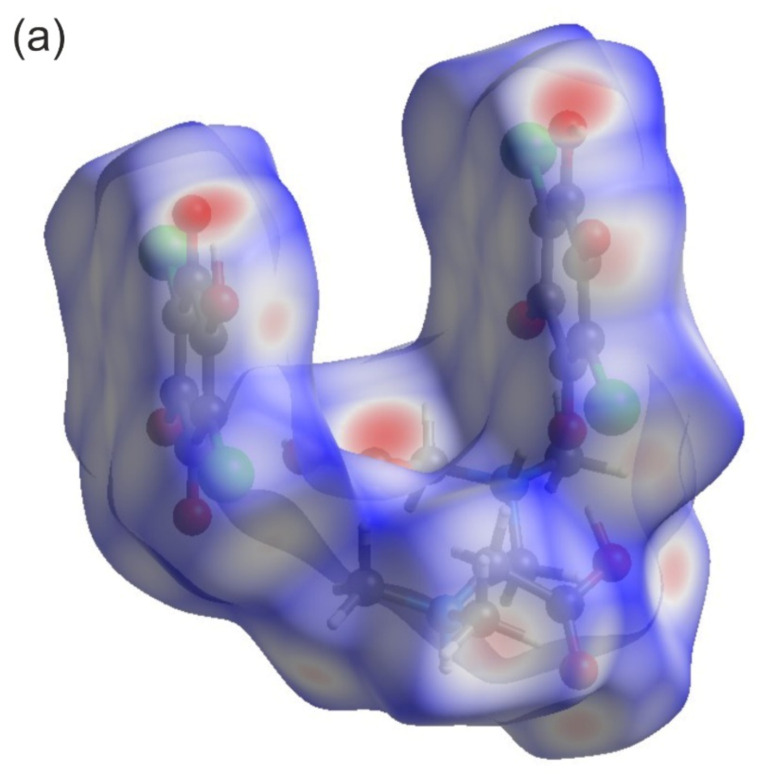

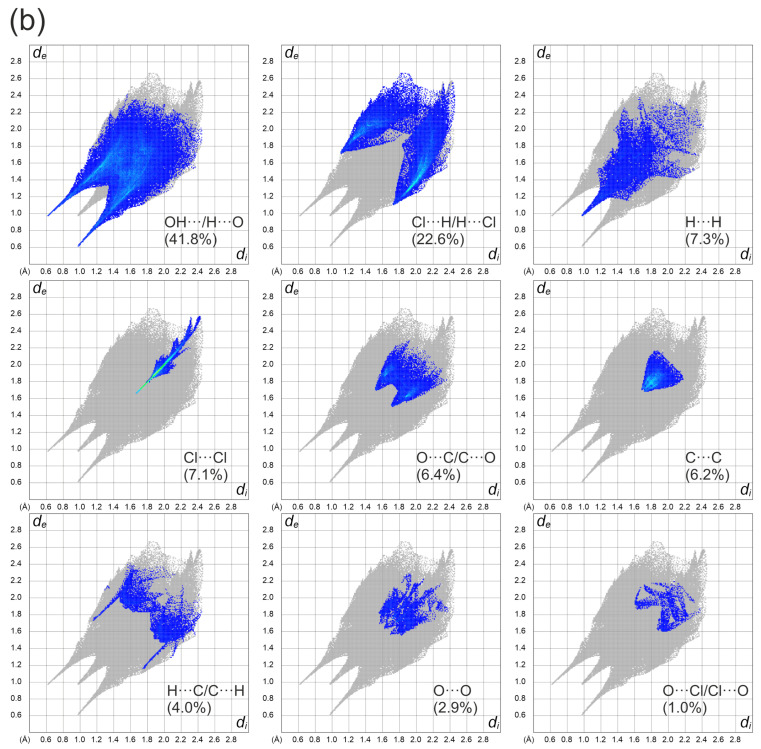
(**a**) Hirshfeld surface analysis for the N,N-dimethylglycine–chloranilic acid co-crystal. Parameters mapped onto the surface *d_norm_*. (**b**) Fingerprint plots generated with relative percentage contributions of particular intermolecular contacts to the Hirshfeld surface area.

Figure 4a depicts a Hirshfeld surface. It was generated using a CrystalExplorer program [41] for room temperature crystal structures. The Hirshfeld surface is an interesting concept, allowing one to visualize the most important interactions that can be found in the studied sample. Many properties can be mapped to such surfaces. We have plotted the so-called *d*_norm_ parameter. It provides information about intermolecular contacts between atoms. If the contacts are closer than the sum of the van der Waals radii of the atoms, the appropriate region of the surface is red-colored. These regions correspond to hydrogen bonds. Blue regions visible on the surface indicate that there is no close contact between atoms inside and outside of the surface. A similar analysis was performed, for example, for the 2,6-dimethylpyrazine and picric acid co-crystal [42]. The other properties that can be mapped onto the surface are the *d*_i_ and *d*_e_ parameters. The *d*_i_ and *d*_e_ parameters describe the distance from the surface to the nearest atom inside or outside the surface, respectively. In Figure 4b, the fingerprint plots (*d*_e_ versus *d*_i_) for the investigated co-crystal are shown. In this picture, contributions from the main interactions were marked together with the percentage of the total area. The nine presented interactions contribute 99.3% of the total area. The C–Cl interactions contribute ~0.8%. This is the smallest contribution and was omitted in the figure. As can be seen in this figure, the O–H interactions contribute 41.8% (Figure 4b). This is the highest contribution presented in the fingerprint plot.

Two characteristic sharp spikes are clearly visible. The second highest area is covered by H–Cl interactions (22.6%). The remaining intermolecular interactions contribute less than 10% of the total surface. During the analysis, it was found that particular interactions contribute to the fingerprint in the following amounts: O–H (41.8%), H–Cl (22.6%), H–H (7.3%), Cl–Cl (7.1%), O–C (6.4%), C–C (6.2%), H–C (4%), O–O (2.9%), O–Cl (1%), C–Cl (0.8%). The other interactions (O–N, H–N, N–N, N–C, N–Cl) are not visible. It can be concluded that hydrogen bonds are the most important interaction found in the studied co-crystal.

### 3.2. NMR Spectroscopy

The structure of the complexation product of chloranilic acid (CLA) and N,N-dimethylglycine (DMG) was investigated in both a DMSO solution and in the solid state. The results of the NMR measurements of substrates and the complexation product are shown in Table 4.

The first problem is the structures of CLA and DMG in both phases. The carbon spectrum of CLA in the DMSO solution only has two signals, one from chlorine-substituted carbon atoms and the second one from atoms connected with oxygen atoms, where two protons in the molecule are distributed over four positions. A completely different situation was found in the CPMAS carbon spectrum of CLA. In this case, four well-separated signals were detected. In the region typical for oxygen bonded to carbon atoms, we have two signals: 176.0 ppm typical for the carbonyl group and 154.9 ppm characteristic of phenol carbon atoms. This indicates that in the solid state the proton exchange process is stopped, probably by intermolecular hydrogen bonds. Two signals from chlorine-substituted carbon atoms suggested that two hydroxyl groups are located in the 1,3 position leading to an unsymmetrical structure, in contrast to the 1,4 symmetrical structure. A similar relation between spectra in the solution and the solid states is observed for the complexation product. Once again, a dynamic averaged structure leading to two signals was found in DMSO. The most interesting finding is the low-field signal δ = 167.9 ppm assigned to the oxygen-substituted sites. This signal is low-field shifted as compared to the CLA spectrum, which suggests that a proton-transfer process from CLA to DMG occurs. Additionally, this signal is very broad (ν _1/2_ = 88 Hz), which shows that the exchange process is relatively slow, close to the coalescence region. In the solid-state spectrum of the complex, four carbon signals in the low-field region were found. The position of those signals suggests that one proton was transferred from CLA to DMG, forming a nonsymmetrical structure with two nonequivalent C=O groups and one C–OH moiety. The averaged chemical shift difference between neutral C=O and deprotonated C–O^–^ sites is close to 10 ppm. This is close to the effect observed in appropriate chemical shifts between CLA and the complex with DMG in the DMSO solution. In reality, this effect is equal to 2 ppm, but we have to remember that it is averaged over four positions. To confirm that consideration, the deprotonation reactions of CLA by tetramethylguanidine (TMG) were performed. The addition of one equivalent of TMG to CLA in the DMSO solution moves the averaged oxygen-bonded carbon atom low-field signal to a value of 167.95 ppm (very close to the position of this signal in the complex with DMG), the next equivalent of TMG moves this signal to 172.4 ppm, indicating further deprotonation reaction. The formation of a 1:1 complex of CLA and DMG in a DMSO solution was confirmed by diffusion measurement. The molecular weight of the expected complex calculated from the diffusion coefficient is M_w_ = 352. The M_w_ value calculated from the molecular formula is 209 + 103 = 312. In this measurement, the calculated M_w_ value of DMSO was 67, while the correct value is 83. The relatively small difference between measured and calculated M_w_ values confirms the reliability of this method.

In essence, the complexation spectral effect should be visible on the NMR signal positions of the DMG molecule. Unfortunately, the situation is much more complicated. First of all, using the diffusion coefficient measurement with the DOSY (Diffusion-Ordered Spectroscopy) method, we found that in the DMSO solution, DMG exists as a dimer (measured M_w_ = 196, calculated 103), whereas in D_2_O it exists as a single molecule (measured M_w_ = 106). In the dimer structure presented in the DMSO, both proton acceptor positions are partially protonated. In the solid state, the C=O signal from DMG is slightly shifted downfield, indicating some deprotonation process on this position as compared to the DMSO solution. Since the chemical shifts of the C=O atom in DMG in both the pure sample and the potential complex are almost the same, we can state that the proton position in both species is the same, which suggests that the proton transfer responsible for the complex formation occurs in the nitrogen atom. Unfortunately, we are not able to confirm this conclusion on the basis of nitrogen chemical shifts. It is well-known that nitrogen chemical shifts of aliphatic amines are almost insensible in the protonation process. Protonation of such functional groups can produce an effect of only a few ppms in both directions; therefore, this spectral parameter is worthless from this point of view.

### 3.3. Thermal Analysis (TG, DSC)

To check the stability of the title compound, thermal analysis (TG, DTG, DSC) was performed.

**Figure 5:**
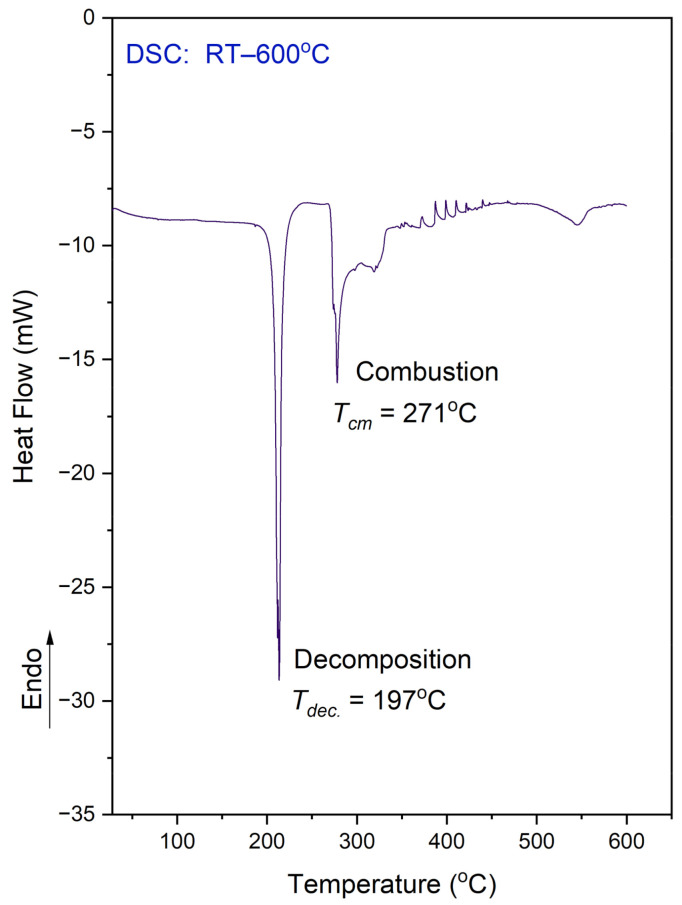
DSC curve for the N,N-dimethylglycine–chloranilic acid co-crystal, registered at a heating rate of 10 °C min^−1^ in the temperature range of 25–600 °C.

Figure 5 shows the DSC results obtained in the temperature range of 25–600 °C for the N,N-dimethylglycine–chloranilic acid (DMG^+^–CLA^−^) co-crystal.

It is worth mentioning that DSC examinations were conducted on a sample that was enclosed in an alumina vessel with a micro hole, whereas the TG analysis was performed on a sample placed in an open pan. The situation of the samples were different therefore the comparison of both results is not straightforward. In the DSC, the released fragment could not be easily removed from the vessel. On the DSC curve one can see a few peaks. The observed features can be explained using a combined approach and taking into account the TG results.

The exothermic peak, presented on the DSC curve at 197 °C, relates to the decomposition process. The sample decomposes without melting. This situation is different from that which was observed for the L leucine–chloranilic acid complex previously studied [14]. The second peak (at 271.0 °C), followed by another (at 320 °C) not-well-resolved and large exothermic peak are most probably connected with the combustion process of the previously released fragments containing carbon, hydrogen, and oxygen. This is a continuous process. Finally, at 546 °C, there is one more anomaly visible, which is smaller than the previous ones.

The DSC measurements were also conducted in the temperature range from 25 °C to −163 °C (on cooling and heating, with a scan rate equal to 10 °C/min). This examination did not reveal any anomaly on the DSC curve. Therefore, there is no phase transition in the solid state within the mentioned temperature region.

**Figure 6:**
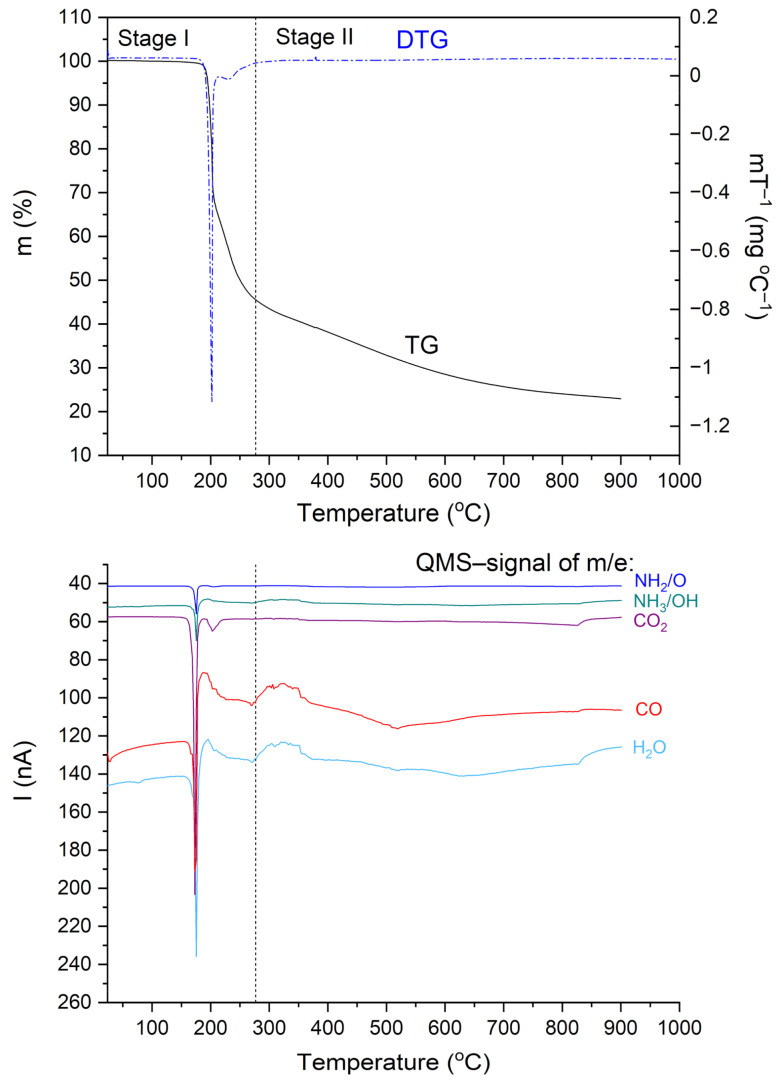
TG, DTG, and QMS curves for the N,N-dimethylglycine–chloranilic acid complex registered at a heating rate of 10 °C min^−1^ in the temperature range of 25–900 °C.

Figure 6 shows TG, DTG, and QMS curves recorded for the N,N-dimethylglycine-chloranilic acid complex. The QMS spectra for m/e = 16, 17, 18, 28, and 44 are shown. These lines correspond to the following fragmentation moieties: NH_2_/O, NH_3_/OH, H_2_O, CO, and CO_2_, respectively. The greatest change in mass occurs at ca. 200 °C. This corresponds very well to the first DSC peak. The fragments described by QMS signals are released and removed from the sample. After further heating, the organic residues are gradually removed. From the results presented above, one can conclude that the decomposition process proceeds in two steps. These results are compatible with the DSC results. In the first step (temperature range 25–277 °C), the complex loses 55.0% of the starting weight. Above 277 °C, further degradation of the sample occurs and a second step can be distinguished. The weight lost was 22.0% and the final mass after the TG experiment (at 900 °C) was 23.0% of the initial mass. The calculated value of the carbon residue (C_6_) is 23.1%.

The thermal decomposition of the investigated complex proceeds through more complicated processes such as decarboxylation, deamination, and condensation reactions. The differential scanning calorimetry (DSC) results show that the decomposition process is more complex than in the case of TG measurements. These differences can be attributed to different measuring conditions. Thermal analysis confirms that the DMG^+^–CLA^−^ co-crystal is stable up to a temperature of 190 °C.

### 3.4. Vibrational Spectra

The investigated N,N-dimethylglycine–chloranilic acid (DMG^+^–CLA^−^) co-crystal contains 120 atoms in a unit cell. Hence, one should expect 360 normal modes. 312 of them describe internal normal vibration modes, 21 describe translation, 24 are rotation modes, and 3 are nonactive acoustic modes. The crystal is centrosymmetric, so the mutual exclusion rule applies. Vibrations active in IR spectroscopy are silent in RS and vice versa. According to the selection rules in the C_i_ factor group, one gets the following irreducible representation: Γ_vib_ = 180A_g_ + 180A_u_ (including acoustic modes). Modes with A_u_ symmetry are active only in IR, while A_g_ modes are active only in Raman spectra for k = 0. Details are described in Table 5.

**Figure 7:**
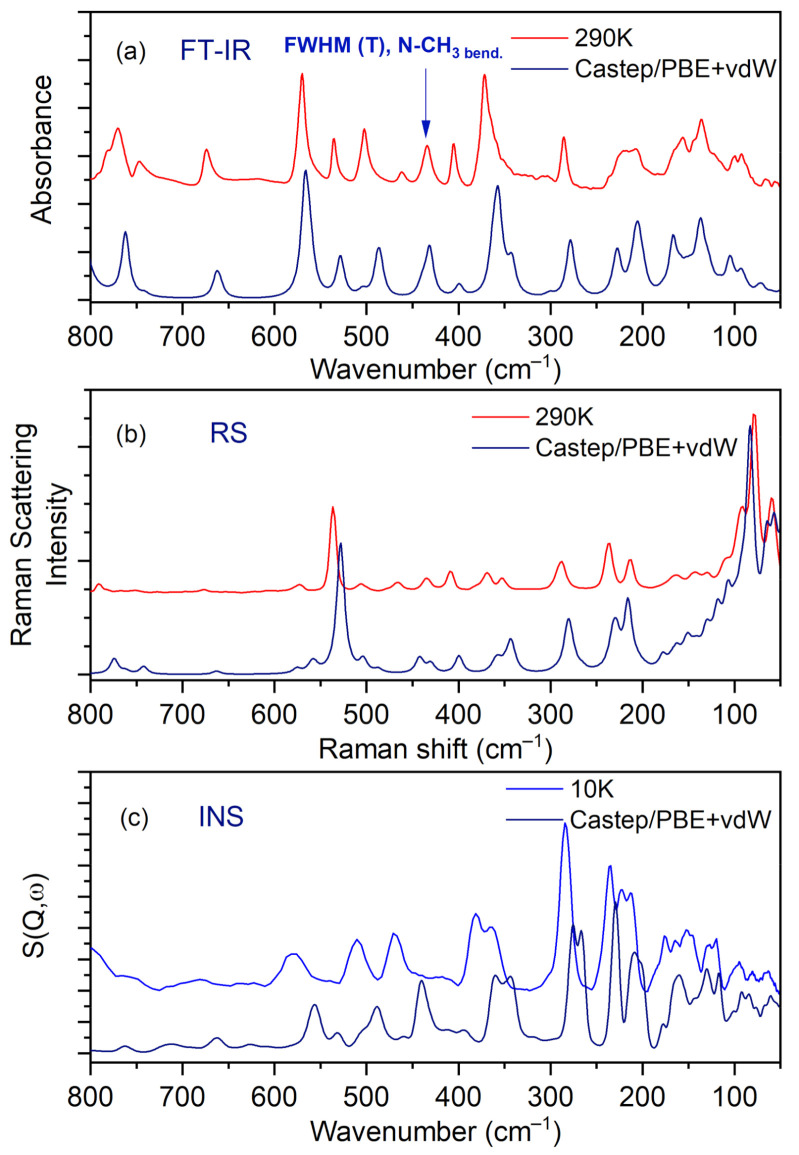
Comparison of experimental and calculated (at PBE + vdW level): (**a**) IR, (**b**) RS, and (**c**) INS spectra for the N,N-dimethylglycine–chloranilic acid complex in the region of 800–50 cm^−1^.

IR, Raman, and INS spectra measured at 290 K, 290 K, and 10 K, respectively, as well as their calculated in periodic boundary condition counterparts (range 800–50 cm^−1^) are presented in Figure 7.

**Figure 8:**
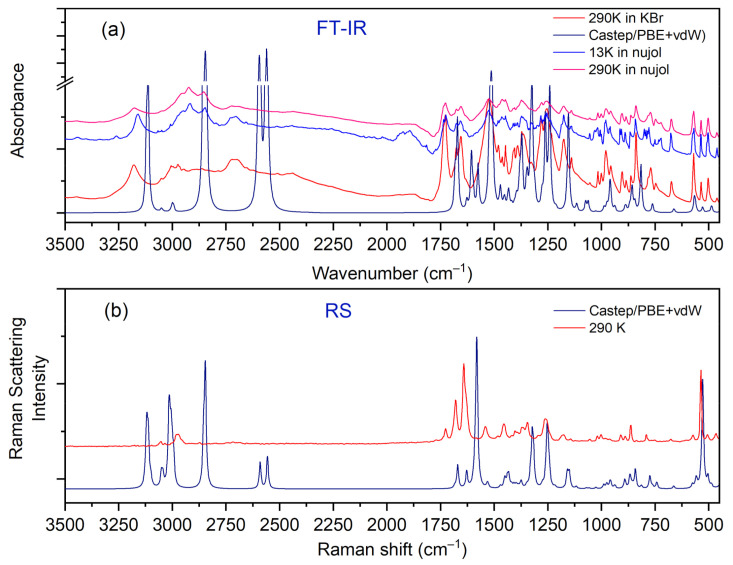
Comparison of experimental and calculated (at PBE + vdW level): (**a**) IR and (**b**) RS spectra for the N,N-dimethylglycine–chloranilic acid complex in the region of 3500–450 cm^−1^.

The most intensive peaks presented in the INS spectrum are connected to torsion vibrations of the methyl CH_3_ groups. These bands are located between 200 and 285 cm^−1^ at the following wavenumbers: 211, 223, 235, and 284 cm^−1^. The calculated values are shifted slightly with respect to the experimental ones and are found at 209, 229, 265, and 276 cm^−1^. Very good agreement between experiment and calculations is evident. The IR and RS spectra in the energy range of 3500–450 cm^−1^ are shown in Figure 8.

Table 6 lists the experimental IR, Raman and INS wavenumbers along with the calculated values. The assignment, which was accomplished based on available literature data [18,19,23,43] and visualization of modes in Jmol software [44], is also provided.

Careful inspection of the middle infrared spectrum reveals that the bands are located on a high background. This background is rather bumpy, not flat. There are three distinct and broad Gauss-like curves illustrating the changes in the background. These peaks are centered at approximately 1300, 1895, and 2722 cm^−1^. Such a picture reassembles the so-called Hadżi trio and is characteristic of systems with strong N^+^–H···O^−^ hydrogen bonds. It is worth noting that similar features were also observed for other complexes containing CLA as one of the components [15,43,45,46].

In the IR experimental spectra at 3179 cm^−1^ (290 K), the O–H stretching vibration modes of CLA molecules are visible. The calculated wavenumber for this vibration is shifted towards the lower values and is equal to 3113 cm^−1^. In the Raman spectrum, we do not observe the band associated with this vibration. A low intensity shoulder band at 3050 cm^−1^ (IR spectrum) is connected to the asymmetrical stretching CH_3_ modes of DMG^+^. The Raman and theoretical bands are almost in the same place. In the region of 3044–2995 cm^−1^ of the calculated spectra, asymmetrical and symmetrical stretching CH_3_ and CH_2_ modes are observed.

In the 2900–2788 cm^−1^ N–H region, stretching vibrations of the aliphatic amino acid (DMG^+^) are visible. The calculated bands are sharp, very intensive, and well-separated; however, in the experimental IR spectra, we can observe a broad band with the shoulder bands. In the IR spectra between 2700 and 2400 cm^−1^ we can observe hydrogen bond vibrations and the O–H stretching vibration of DMG^+^. In the RS spectrum, there are no visible bands in this region (see Figure 8). A very broad peak (IR spectrum) with its maximum at 1874 cm^−1^ is connected to the overtone (CH_2 bend._) and summation (CH_2 bend._ + CH_2 wagg._) vibration modes. We do not observe these bands in the calculated spectra. In the IR experimental spectrum, the intensive peak at 1523 cm^−1^ relates to the bending NH^+^, COH modes, whereas in the experimental RS spectra this band is of low intensity. Near 1480–1400 cm^−1^ bending CH_3_, NH^+^, CH_2_ modes are observed (from DMG^+^ cation). This region (both IR and RS) is very well-represented by computations. Intensive bands between 1255 and 1000 cm^−1^ were interpreted, among others, as rocking CH_3 rock._ (DMG^+^) modes of N,N-dimethylglycine. The calculated bands are sharp and shifted towards lower wavenumbers (see Figure 8). In the wavenumber range of 1000–600 cm^−1^ in the IR and RS spectra, we can observe the ring deformation, OH torsion, and COH bending of chloranilic acid anion, as well as C–N stretching, C–C stretching, CH_2_ rocking, and COOH bending of the N,N-dimethylglycine cation modes. In this range, there is a good agreement between experimental and predicted IR and RS spectra.

In the wavenumber range of 600–50 cm^−1^, N–CH_3_ bending, CH_2_ rocking, C–C–N bending, C–N–C bending, and CH_3_ torsion modes for N,N-dimethylglycine cation are observed. For the chloranilic acid anion in this same region, ring deformation, COH bending, C=O wagging, and ring torsion modes are also observed. Lattice vibrations are active below 130 cm^−1^.

### 3.5. Temperature-Dependent IR Spectroscopy

**Figure 9:**
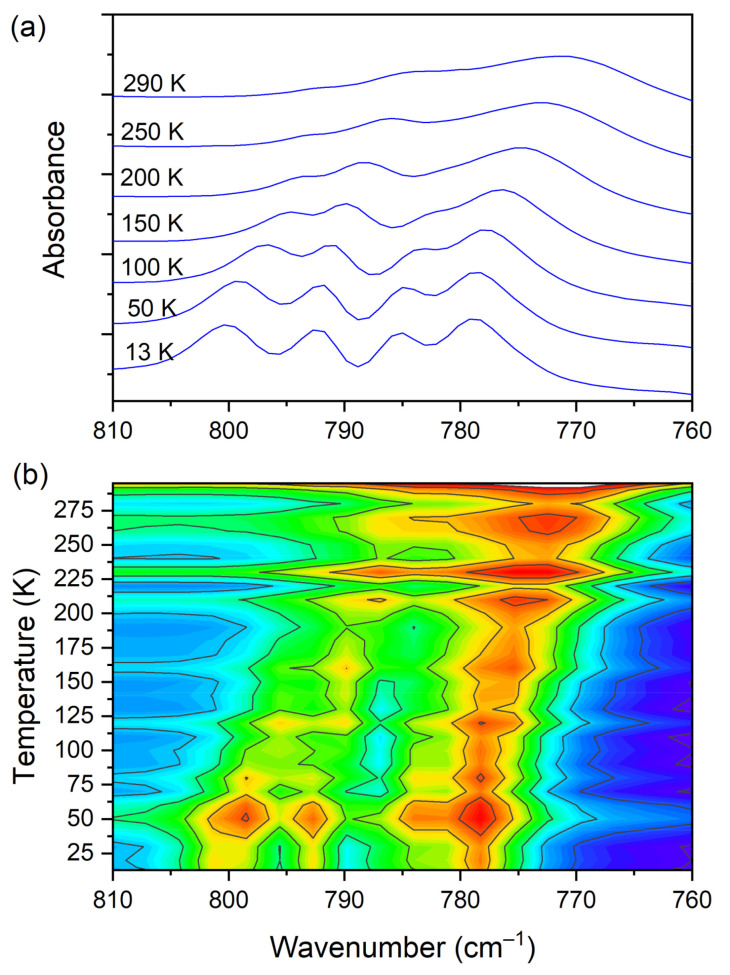
(**a**) IR spectra in the wavenumber range of 810–760 cm^−1^ during cooling of the N,N-dimethylglycine–chloranilic acid co-crystal; (**b**) contour plots of IR spectra in the C−Cl _str._ (CLA^−^) vibration region.

The IR spectra (both FIR and MIR) were measured for the DMG^+^–CLA^−^ complex at various temperatures. The investigated complex does not exhibit any phase transition in the temperature range of 300–100 K. Hence, for most of the registered bands, no spectacular changes were observed. One can notice the narrowing (increasing intensity) of the peaks during the cooling of the sample. This is natural behavior. The position of the peaks does not change as well. However, in a few wavenumber regions, more significant changes can be noticed. Among the most interesting, four ranges can be highlighted. These are the following: 810–760, 1045–990, 2800–1800, and 3200–3120 cm^−1^. The first mentioned region is covered by C−Cl _str._ (CLA^−^) vibrations and is displayed in Figure 9.

**Figure 10:**
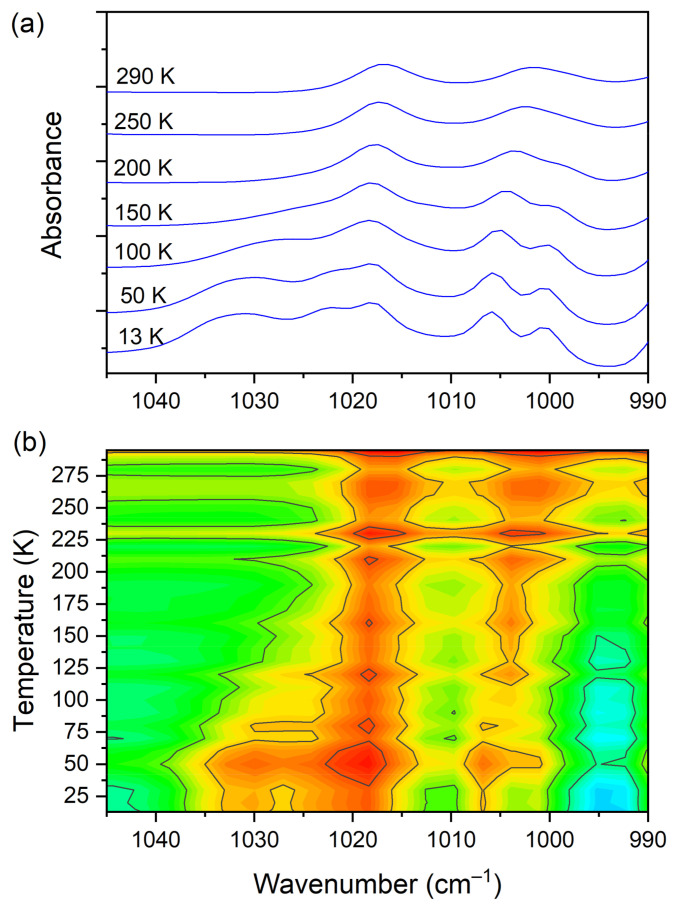
(**a**) IR spectra in the wavenumber range of 1045–990 cm^−1^ during cooling of the N,N-dimethylglycine–chloranilic acid co-crystal; (**b**) contour plots of IR spectra in the C−N _str._, CH_3 rock._, CH_2 rock._ (DMG^+^) vibration regions.

**Figure 11:**
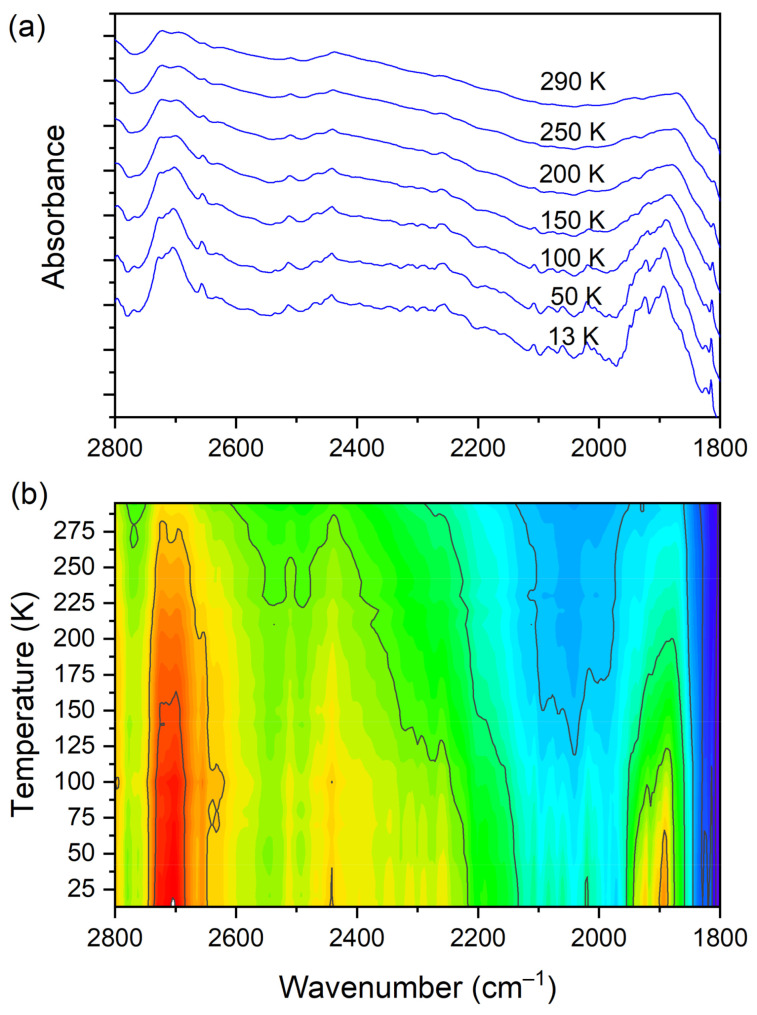
(**a**) IR spectra in the wavenumber range of 2800–1800 cm^−1^ during cooling of the N,N-dimethylglycine–chloranilic acid co-crystal; (**b**) contour plots of IR spectra in the overtone (C–H_2 bend._) and summation (CH _bend._ + CH _wagg._) regions, and O–H stretching modes (DMG^+^).

**Figure 12:**
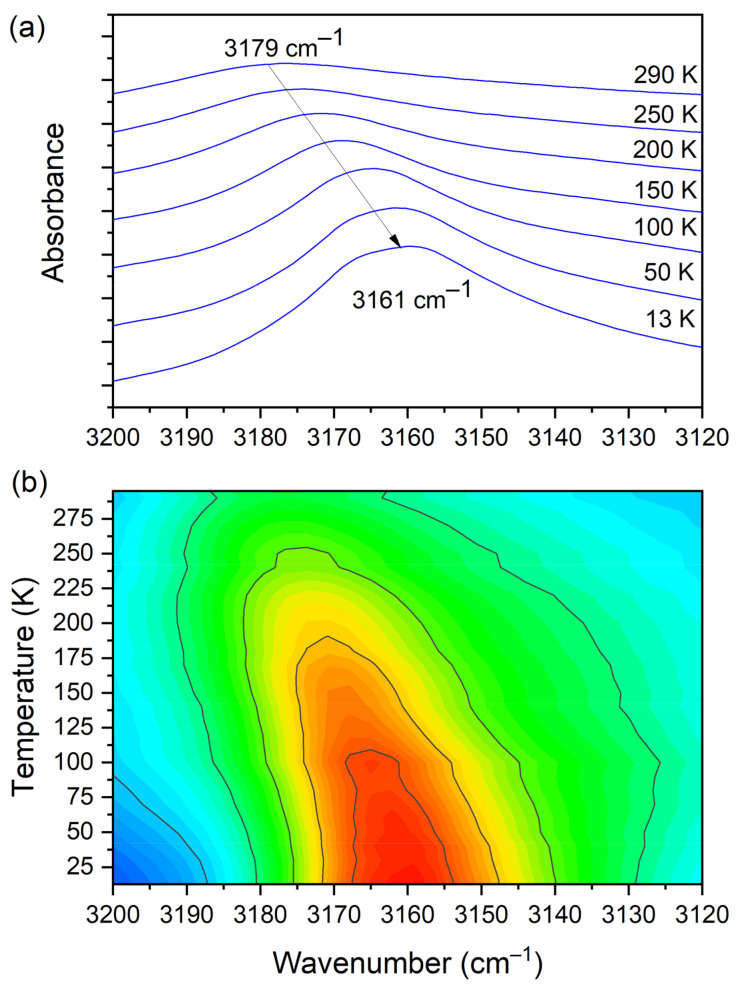
(**a**) IR spectra in the wavenumber range of 3200–3120 cm^−1^ during cooling of the N,N-dimethylglycine–chloranilic acid co-crystal; (**b**) contour plots of IR spectra in the H-bonding region (O–H vibration from CLA acid).

At room temperature one can see two very broad and diffused bands. During cooling, these two maxima move away from each other. The initially hidden bands emerged from the spectrum and four well resolved peaks are clearly visible at the lowest temperature. The shifting and appearing of these bands are nicely visible in the contour plot figure. In the second region, vibrations connected with C−N _str._, CH_3 rock._, CH_2 rock._ (DMG^+^) are visible. Here we have a very similar picture. At room temperature two bands can be detected, but at 13 K, five spectral features are present. These changes are depicted in Figure 10. The bands visible in the third wavenumber range (Figure 11) are located on a relatively high background. In this region, the bands are related to overtone (C–H_2 bend._) and summation (CH _bend._ + CH _wagg._) vibrations modes and O−H stretching modes (DMG^+^). Very interesting changes are visible in the fourth mentioned region (Figure 12).

In this range, the O–H vibration of CLA acid appears. These atoms are engaged in the formation of hydrogen bonds: O3C–H3C···O1B, O3D–H3D···O1A. Therefore, this vibration is a sensitive indicator of the strength of the formed hydrogen bonds. At high temperatures, the O–H stretching vibration is very broad and blurred. During cooling, one can observe not only a narrowing of this peak but also a significant (18 cm^−1^) shift towards lower wavenumbers. The center of this peak moves from 3179 cm^−1^ at 290 K to 3161 cm^−1^ at 13 K. Such a meaningful shift is, without doubt, connected to an increase in the strength of the hydrogen bonds. The distance between H and O in O–H(CLA^−^)···O(DMG^+^) (~2.1 Å at room temperature) is reduced (see Figure 1 and Figure 3). The hydrogen pattern in this complex is complicated. Besides the formerly described interactions, there are hydrogen bonds formed between: N1B–H1B(DMG^+^)···O6C(CLA^−^) (H1B···O6C distance = 2.032 Å, angle N–H–O = 160.9°), N1B–H1B(DMG^+^)···O1C(CLA^−^) (H1B···O1C distance = 2.436 Å, angle N–H–O = 123.3°), N1A–H1A(DMG^+^)···O6D(CLA^−^) (H1A···O6D distance = 1.994 Å, angle N–H–O = 158.3°) and N1A–H1A(DMG^+^)···O1D(CLA^−^) (H1A···O1D distance = 2.393 Å, angle N–H–O = 121.2°). As one can see here, we have two rather short hydrogen bonds (~2 Å) and two slightly longer ones (~2.4 Å). However, the dynamical properties of the N–H···O hydrogen bonds are different in comparison with O–H···O interactions. The bands connected with N–H vibrations do not change their position in IR spectra. This interaction is strong at 293 K and the decrease in temperature does not have a strong impact on the N–H vibration.

**Figure 13:**
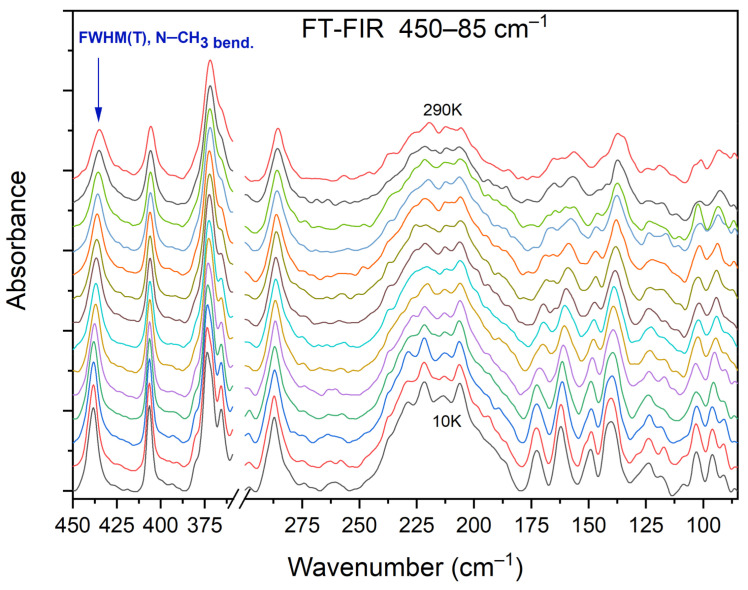
Selected FIR spectra in the wavenumber range of 450–80 cm^−1^ during cooling of the N,N-dimethylglycine–chloranilic acid co-crystal from 290 K to 10 K, with the step 10 K.

Figure 13 shows the selected FIR spectra of the DMG^+^–CLA^−^ co-crystal, within the wavenumber range of 450–80 cm^−1^, registered during the cooling of the sample from 290 K to 10 K. One band (at 434 cm^−1^) was chosen for further analysis.

This peak relates to the N–CH_3_ bending vibration, and its width decreases during cooling. We performed a band-shape analysis for this band. For such an analysis to be possible, three conditions must be met. The chosen peak should not overlap with others. This suggests that it should be isolated in a sufficiently wide range of wavenumbers. The second condition states that in the analyzed vibration, only one motion of a particular group should be involved. The third and final requirement is that the intensity of the selected peak should not be too low. Looking at Figure 13, one can see that all these conditions were fulfilled: the peak is isolated (both in terms of energy and mode involved) and its intensity is considerable.

During routine data analysis, the stress is usually placed on two spectral features. One can be interested in where the particular band is located on a wavenumber scale and what its intensity is. However, some interesting information are likewise encoded in the width of the peak. The reorientational motion of molecular groups is reflected in the peak width. On cooling, the width of the band decreases. Without going into details, one can say that two relaxation mechanisms may be distinguished: the vibrational and the reorientational relaxation. The band shape analysis is a tool used to extract information regarding the molecular reorientational motion. It is known that the width of the peak (expressed in cm^−1^) is inversely proportional to the reorientational correlation of time τ_c_. Optical spectroscopies such as IR and RS give access to motions, which occur within a 10^−13^–10^−11^ s time scale. This is the so-called time window and it is characteristic of a particular method. The methodology is as follows: One needs to collect vibrational spectra from a wide temperature range. The chosen band is approximated by a bell-shaped curve (Lorentz or Gaussian function), which is fitted to the experimental data in the analyzed region. Next, the temperature dependency of full-width-at-half-maximum (FWHM) is investigated. The theoretical considerations for the order–disorder transition mechanism are given in Ref [47,48].

One of the crucial parameters is the reorientational correlation time τ_c_. It can be described by Equation (2):

(2) $$\tau_{c}=\tau_{\infty }\cdot \exp\left(\frac{E_{a}}{RT}\right),$$

where the molecule residues for a τ_c_ time in one potential well and jumps (infinitely fast) to another potential well. The preexponential factor τ_∞_ denotes the relaxation time at an infinite temperature. Jumps occur through the potential energy barrier *E*_a_. *R* is the gas constant.

Temperature dependence of the FWHM can be expressed by Equation (3) [47,48,49]:

(3) $$\mathrm{FWHM}\left(T\right)\;=\;\left(a+b\cdot T\right)\;+\;c\cdot \exp\left(-\frac{E_{a}}{RT}\right),$$

where the parameters a, b, c, and *E_a_* are obtained during the least square fitting procedure. This formula is fitted to the experimental data. In Equation (3), two parts are clearly visible. The linear part relates to the above-mentioned vibrational relaxation process. It does not strongly depend on temperature. The exponential term describes the reorientational motion.

In the studied system, methyl groups belonging to N,N-dimethylglycine may reorientate and can be responsible for the narrowing of some bands on cooling.

Analysis of the temperature dependency of the full-width-at-half-maximum (FWHM) was carried out for the band at 434 cm^−1^, associated with the N–CH_3_ bending mode.

**Figure 14:**
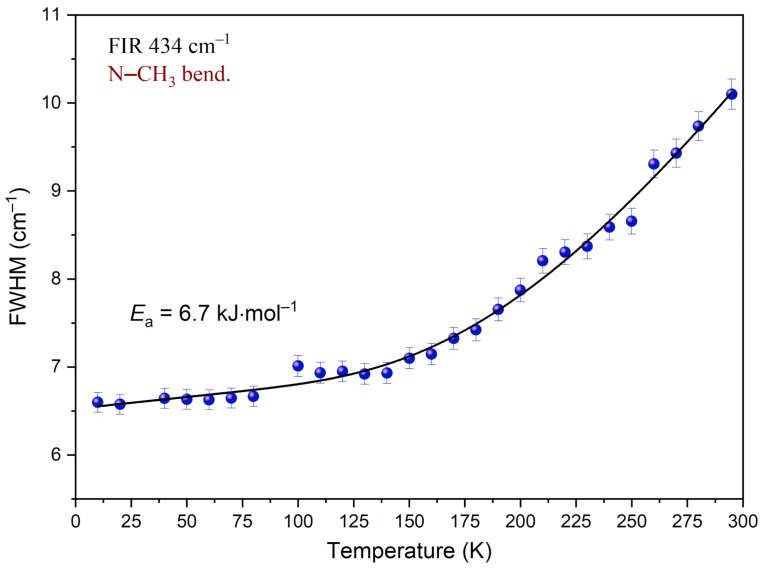
Temperature evolutions of FWHM of the band at 434 cm^−1^ associated with the N–CH_3_ bending mode. The solid line represents fitting of Equation (3) to the experimental data.

Figure 14 shows the changes of peak width while the temperature decreases. Inspection of this figure reveals that FWHM decreases when the sample is cooled. In the temperature region of 295–140 K, this change is well-described by an exponential function. Further cooling of the sample has a much smaller impact on FWHM. The dependency below 140 K is well-described by a linear function. The function reaches a plateau. It indicates that considering the time window, the motion of the N–CH_3_ group is slowed down. Equation (3) was fitted to the experimental data in the temperature region of 290–10 K. Parameters obtained during this procedure are gathered in Table 7. As a result of the performed analysis, the mean value of the activation energy *E_a_* was estimated. *E_a_* is equal to 6.7 kJ mol^‒1^. Comparing this value with other results obtained for compounds containing methyl groups, one can see that they fall in the same range. Based on the band shape analysis performed for the N,N-dimethylglycine–chloranilic acid co-crystal, it can be said that the fast N–CH_3_ group reorientation slows down below 140 K and the correlation time τ_c_ is greater than 10^−11^ s.

## 4. Conclusions

At room temperature, the new co-crystal N,N-dimethylglycine–chloranilic acid crystallizes in a triclinic crystal system within space group No.2 = P-1, with four molecules in the unit cell. The following unit cell constants were determined: a = 9.6199(4) Å, b = 12.0092(5) Å, c = 12.8763(6) Å, α = 97.749(4)°, β = 110.173(4)°, and γ = 111.415(4)°.

From NMR and X-ray measurements, it was found that N,N-dimethylglycine exists in the cation form (protonated form) and chloranilic acid exists in the anion form (deprotonated form).

Cations and anions form a layered structure. They are bonded to each other by a series of intermolecular N^+^–H···O^–^ and O^+^–H···O^–^ hydrogen bonds. The N–H amino groups are involved in bifurcated hydrogen bonds, with two carbonyl oxygen atoms of neighboring chloranilic acid molecules.

X-ray diffraction and IR investigation confirm the presence of a strong N^+^–H···O^–^ hydrogen bond in the studied co-crystal. In the IR spectrum, one can observe a broad, continuous absorption with a well-separated and so-called Hadżi trio, typical of strong hydrogen bonds with peaks at approximately 1300, 1895, and 2722 cm^−1^.

Thermal analysis showed that the investigated complex is stable up to 190 °C. It subsequently decomposes, and combustion processes are observed. The normal modes were described and analyzed as a function of temperature. During the cooling of the complex from room temperature to 13 K, we observe quite significant changes in the bands related to C–H_2 bend._, summation (CH _bend._ + CH _wagg._), and O–H vibrations of the CLA anion.

The experimental IR, RS, and INS spectra were compared with DFT, calculated for periodic boundary conditions using the CASTEP code; a good agreement was consequently achieved. The N–CH_3_ groups perform fast (τ_R_ ≈ 10^–11^–10^–13^ s) stochastic reorientational motions with the average energy of *E_a_* ≈ 6.7 ± 0.6 kJ mol^−1^. However, below the temperature of 140 K, the fast stochastic reorientations become slow enough that they are not registered by optical spectroscopy.

## Figures and Tables

**Table 1:** Crystal data and structure refinement for N,N-dimethylglycine–chloranilic acid co-crystal.

Empirical Formula	C_10_H_11_Cl_2_NO_6_
Formula weight	312.10
Temperature	293(2) K
Wavelength	1.54184 Å
Crystal system	Triclinic
Space group	P-1
Unit cell dimensions	a = 9.6199(4) Å, α = 97.749(4)°.
	b = 12.0092(5) Å, β = 110.173(4)°.
	c = 12.8763(6) Å, γ = 111.415(4)°.
Volume	1240.68(10) Å^3^
Z	4
Density (calculated)	1.671 Mg/m^3^
Absorption coefficient	4.960 mm^−1^
F(000)	640
Crystal size	0.2 × 0.2 × 0.03 mm^3^
Theta range for data collection	5.16 to 71.16°.
Index ranges	−11 ≤ h ≤ 10, −14 ≤ k ≤ 14, −15 ≤ l ≤ 15
Reflections collected	15,069
Independent reflections	4409 (R(int) = 0.1036)
Completeness to theta = 71.16°	91.9%
Refinement method	Full-matrix least-squares on F^2^
Data/restraints/parameters	4409/0/361
Goodness-of-fit on F^2^	0.980
Final R indices (I > 2σ(I))	R1 = 0.0471, wR2 = 0.1233
R indices (all data)	R1 = 0.0553, wR2 = 0.1283
Largest diff. peak and hole	0.546 and −0.303 e·Å^−3^

**Table 2:** Selected bond lengths, bond and torsion angles of N,N-dimethylglycine–chloranilic acid (DMG^+^–CLA^−^) co-crystal at room temperature.

Coordinates	Experimental X-ray	Calculated CASTEP/PBE + vdW	Coordinates	Experimental X-ray	Calculated CASTEP/PBE + vdW
Bond lengths (Å)			N1A–C4A–H4A1	109.5(1)	107.8
C1A–C2A	1.512(3)	1.521	N1A–C4A–H4A3	109.5(1)	109.2
C1C–C2C	1.466(4)	1.452	N1B–C3B–H3B2	109.5(1)	108.6
C1D–C2D	1.458(4)	1.449	O1A–C1A–O2A	121.8(2)	121.8
C2A–H2A1	0.971	1.097	O1B–C1B–C2B	122.2(2)	122.6
C2A–H2A2	0.970	1.097	O1D–C1D–C6D	118.8(2)	118.2
C2C–C3C	1.332(4)	1.363	O2A–C1A–C2A	116.1(2)	115.9
C3A–H3A1	0.960	1.091	O3D–C3D–C2D	122.5(2)	123.0
C3C–C4C	1.512(4)	1.513	O4D–C4D–C3D	115.6(2)	115.0
C4A–H4A1	0.960	1.092	O6D–C6D–C5D	126.6(2)	126.1
C4C–C5C	1.421(4)	1.422	Torsion angles (°)		
C5C–C6C	1.371(4)	1.395	H2A–O2A–C1A–O1A	−180(3)	179.1
Cl2C–C2C	1.723(3)	1.731	H2A–O2A–C1A–C2A	1(3)	−0.2
Cl5D–C5D	1.731(3)	1.737	H1A–N1A–C2A–H2A2	76	73
N1A–H1A	0.860(4)	1.061	H1A–N1A–C3A–H3A1	−62	−57
N1A–C3A	1.490(4)	1.505	H1A–N1A–C3A–H3A2	178	−176.1
N1A–C4A	1.484(3)	1.504	H1A–N1A–C3A–H3A3	58	63.7
N1A–C2A	1.483(2)	1.498	C2A–N1A–C4A–H4A1	−179.7	171.9
O1A–C1A	1.208(4)	1.225	C2A–N1A–C4A–H4A2	60.3	52.5
O2A–H2A	0.820(4)	1.032	C3A–N1A–C4A–H4A2	−174.7	177.4
O2A–C1A	1.303(2)	1.320	C3A–N1A–C4A–H4A3	65.4	57.6
O2B–H2B	0.730(4)	1.030	O2A–C1A–C2A–N1A	−173.9(2)	−172.6
O3D–C3D	1.325(3)	1.319	O2A–C1A–C2A–H2A1	52.8	−50.8
O4C–C4C	1.227(3)	1.243	H2B–O2B–C1B–C2B	−3(3)	−0.8
O3D–H3D	0.840(4)	1.002	C3B–N1B–C2B–C1B	−162.1(2)	−161.2
Bond angles (°)			C3B–N1B–C2B–H2B1	76.7	75.5
C1A–C2A–H2A1	109.2(1)	111.1	C4B–N1B–C2B–H2B1	179.6(2)	177.7
C1B–C2B–H2B2	109.1(1)	109.0	C2B–N1B–C3B–H3B1	180	173.4
C1C–C2C–C3C	120.7(2)	120.2	C2B–N1B–C3B–H3B2	59.8	53.9
C1C–C6C–C5C	117.8(2)	118.4	H1B–N1B–C4B–H4B2	179	−175.3
C2A–N1A–C3A	112.4(2)	112.2	C2B–N1B–C4B–H4B3	−59.8	−55.6
C2A–N1A–C4A	111.0(2)	111.1	O1B–C1B–C2B–N1B	4.3(4)	4.3
C2C–C1C–C6C	118.2(2)	118.3	O2B–C1B–C2B–N1B	−176.2(2)	−176.4
C2B–N1B–C4B	110.4(2)	110.1	H3C–O3C–C3C–C4C	0(3)	−3.7
C3C–C4C–C5C	117.2(2)	117.8	C6C–C1C–C2C–Cl2C	176.7(2)	177.1
C4C–C5C–C6C	123.7(2)	122.4	O1C–C1C–C6C–C5C	−176.2(2)	−175.5
Cl2C–C2C–C1C	117.5(2)	118.6	C1C–C2C–C3C–O3C	−179.0(2)	−178.6
Cl2C–C2C–C3C	121.7(2)	121.0	O3C–C3C–C4C–O4C	4.1(3)	4.2
H1A–N1A–C2A	107.0(3)	108.0	O4C–C4C–C5C–Cl5C	−4.7(4)	−5.3
H1A–N1A–C4A	108.0(3)	106.2	C3C–C4C–C5C–C6C	−6.1(4)	−5.7
H2A–O2A–C1A	110.0(3)	114.5	O1D–C1D–C2D–Cl2D	0.7(4)	0.2
H2B–O2B–C1B	118.0(3)	113.9	O1D–C1D–C2D–C3D	−178.3(3)	−178.3
H2B1–C2B–H2B2	107.8(1)	107.2	O1D–C1D–C6D–C5D	−179.9(2)	−179.5
H3A1–C3A–H3A2	109.4(1)	109.4	Cl2D–C2D–C3D–C4D	−179.7(2)	−179.9
H3C–O3C–C3C	107.0(3)	108.0	C1D–C2D–C3D–C4D	−0.7(4)	−1.6
H4A1–C4A–H4A3	109.5(1)	111.0	C2D–C3D–C4D–C5D	−2.0(4)	−0.5
H4B2–C4B–H4B3	109.5(1)	110.1	C3D–C4D–C5D–C6D	4.0(4)	2.9
N1A–C2A–H2A1	109.2(1)	108.8	C4D–C5D–C6D–O6D	176.4(2)	176.9
N1A–C3A–H3A1	109.5(1)	108.8	C4D–C5D–C6D–C1D	−3.0(4)	−3.0

**Table 3:** Hydrogen bonds for the N,N-dimethylglycine–chloranilic acid co-crystal (Å and °).

D–H···A	d(D–H)	d(H···A)	d(D···A)
O(2A)–H(2A)···O(6C)	0.82(3)	1.76(3)	2.555(2)
N(1A)–H(1A)···O(1D)#1	0.86(3)	2.39(3)	2.933(2)
N(1A)-H(1A)···O(6D)#1	0.86(3)	1.99(3)	2.813(2)
O(2B)-H(2B)···O(6D)#2	0.73(3)	1.85(4)	2.578(2)
N(1B)-H(1B)···O(1C)	0.83(3)	2.43(3)	2.971(2)
N(1B)-H(1B)···O(6C)	0.83(3)	2.03(3)	2.826(2)
O(3C)-H(3C)···O(1B)#3	0.87(4)	2.11(4)	2.810(2)
O(3D)-H(3D)···O(1A)#4	0.84(4)	2.15(4)	2.765(2)

Symmetry transformations used to generate equivalent atoms: #1, −x + 1, −y + 1, −z + 2, #2, −x, −y + 1,−z + 2, #3, −x, −y + 1,−z + 1, #4, x, y + 1, z.

**Table 4:** NMR data for the N,N-dimethylglycine–chloranilic acid co-crystal (ppm).

Reagents Used and Measurement Conditions	C=O	C–OH	C–O	C–Cl	CH_3_	CH_2_	COOH	N
CLA in DMSO	165.9			110.01				
DMG in DMSO					43.8 **2.59**	60.8 **3.24**	167.2	−345.2
CLA solid state CPMAS	176.0 ^#^	154.9 ^#^		110.8 114.3				
DMG solid state CPMAS					41.9, 43.3, 44.1, 44.5	58.7, 59.3	169.4, 170.97	−342.4
CLA + DMG Complex DMSO	167.9 ν _1/2_ = 88 Hz			105.1	43.75 **2.80**	56.97 **4.04**	168.0 ν _1/2_ = 1 Hz	−344.1
CLA + DMG Complex CPMAS	178.7 ” 170.1 *”	154.7 ”	164.5 ”	110.0 106.3	45.7	60.1	170.1 *	−342.2
CLA + TMG 1:1 in DMSO	167.95			105.3				
CLA + double excess of TMG in DMSO	172.4			105.4				
Dimethylglycine In DMSO					43.8 **2.59**	60.8 **3.24**	167.2	−345.2
Dimethylglycine + TMG in DMSO					45.76 **2.10**	65.25 **2.56**	173.0	−357.2
N-dimethylglycine D_2_O					43.45 **2.70**	59.82 **3.58**	170.25	−342.1
N-dimethylglycine in D_2_O + HClO_4_					43.73 **2.84**	57.33 **3.93**	168.13	−344.8

^#^—avaraged value 165.45 ppm. ”—averaged value 167 ppm. *—overlapped signals (double intensity). data in bold and underlined: proton chemical shifts. ν _1/2_ half height line width.

**Table 5:** Classification of the fundamental modes (**k** = 0) for the N,N-dimethylglycine –chloranilic acid (DMG^+^–CLA^−^) co-crystal.

UCG	Lattice Modes			Internal Modes		Selection Rules	
C_i_	Ac.	Trans.	Rot.	DMG^+^	CLA^−^	IR	Raman
A_g_		12	12	90	66		x^2^, y^2^, z^2^, xy, xz, yz
A_u_	3	9	12	90	66	x, y, z	

UCG—unit cell (factor) group; Ac.—acoustic modes; Trans.—translation modes; Rot.—rotation modes.

**Table 6:** Observed and calculated frequencies for the N,N-dimethylglycine–chloranilic acid (DMG^+^–CLA^−^) co-crystal (cm^−1^). The structure is centrosymmetric; thus, vibrations active in IR are not active in Raman and vice versa.

Experimental			Calculated	Approximate Assignments
IR (290 K)	Raman (290 K)	INS	CASTEP/PBE + vdW	
			3122(IR) 3126(R)	CH_3 str. asym._ (DMG^+^)
			3120(IR) 3120(R)	CH_3 str. asym._ (DMG^+^)
			3119(IR) 3119(R)	CH_3 str. asym._ (DMG^+^), O–H _str._ (CLA^−^)
			3118(IR) 3118(R)	CH_3 str. asym._ (DMG^+^)
3179			3113(IR) 3112(R)	O–H _str._ (CLA^−^)
			3102(IR) 3102(R)	CH_3 str. asym._ (DMG^+^)
3050	3051		3052(IR) 3052(R)	CH_2 str. asym._ (DMG^+^)
	3032		3044(IR) 3044(R)	CH_2 str. asym._ (DMG^+^)
			3018(IR) 3017(R)	CH_3 str. sym._ (DMG^+^)
			3013(IR) 3013(R)	CH_3 str. sym._, CH_2 str. asym._ (DMG^+^)
			3006(IR) 3006(R)	CH_3 str. sym._ (DMG^+^)
3006 2996			3000(IR) 3000(R)	CH_2 str. sym._ (DMG^+^)
2974 2947	2976		2995(IR) 2995(R)	CH_2 str. sym._ (DMG^+^)
2911 2887			2857(IR) 2855(R)	N–H _str._ (DMG^+^)
2863 2840	2874		2846(IR) 2846(R)	N–H _str._ (DMG^+^)
2788				N–H _str._ (DMG^+^)
2722 2698	2720		2595(IR) 2592(R)	O–H _str._ (DMG^+^)
2654 2626			2562(IR) 2558(R)	O–H _str._ (DMG^+^)
2510 2468				O–H _str._ (DMG^+^)
2441				O–H _str._ (DMG^+^)
1994				C–H_2 bend._ overtone
1874				CH_2 bend._ + CH_2 wagg._ summation
1734			1682(IR) 1684(R)	C=O _str._, NH^+^ _bend._, COH _bend._ (DMG^+^)
1727	1726		1671(IR) 1670(R)	C=O _str._, NH^+^ _bend._, COH _bend._ (DMG^+^, CLA^−^)
			1629(IR) 1629(R)	C=O _str._, C−C _str._, COH _bend._ (CLA^−^)
			1627(IR) 1628(R)	C=O _str._, C−C _str._, COH _bend._ (CLA^−^)
1676	1679		1606(IR)	C=O _str._, C−C _str._, COH _bend._ (CLA^−^), NH^+^ _bend._ (DMG^+^)
1655	1640		1575(IR) 1581(R)	C=O _str._, C−C _str._, COH _bend._ (CLA^−^)
1523	1541 1582		1515(IR) 1529(R)	NH^+^ _bend._ (DMG^+^), C−C _str._, COH _bend._ (CLA^−^, DMG^+^)
1480	1483		1470(IR) 1470(R)	CH_3 bend._, NH^+^ _bend._ (DMG^+^)
1464			1453(IR) 1453(R)	CH_3 bend._, NH^+^ _bend._ (DMG^+^)
			1435(IR) 1435(R)	CH_3 bend._, NH^+^ _bend._ (DMG^+^)
1447	1455		1433(IR) 1433(R)	CH_3 bend._, NH^+^ _bend._ (DMG^+^)
			1431(IR) 1432(R)	CH_3 bend._, NH^+^ _bend._ (DMG^+^)
			1399(IR) 1399(R)	CH_3 bend._, NH^+^ _bend._, CH_2 bend._ (DMG^+^)
	1416		1396(IR) 1396(R)	CH_3 bend._, NH^+^ _bend._, CH_2 bend._ (DMG^+^)
1407	1403		1371(IR) 1372(R)	CH_3 bend._, NH^+^ _bend._, CH_2 wagg._, COH _bend._ (DMG^+^)
	1390		1351(IR) 1352(R)	CH_2 wagg._, COH _bend._ (DMG^+^)
1391			1346(IR) 1347(R)	CH_2 wagg._, COH _bend._ (DMG^+^, CLA^−^), C−C _str._ (CLA^−^)
1372			1324(IR) 1324(R)	C=O _str._, C−C _str._, COH _bend._ (CLA^−^)
1363	1345 1365		1315(IR) 1318(R)	C=O _str._, C−C _str._, COH _bend._ (CLA^−^)
1328			1277(IR) 1276(R)	CH_2 twist._ (DMG^+^), COH _bend._, CH_3 rock._(CLA^−^)
1279	1294		1262(IR) 1262(R)	C−C _str._, COH _bend._ (CLA^−^), CH_2 wagg._ (DMG^+^)
			1254(IR) 1251(R)	C−C _str._, C−O _str._ (CLA^−^)
			1248(IR) 1247(R)	C−C _str._, C−O _str._ (CLA^−^), CH_2 wagg._, C−O _str._ (DMG^+^)
1255	1259		1243(IR) 1241(R)	C−C _str._, COH _bend._ (CLA^−^), CH_2 rock._, CH_3 rock._ (DMG^+^)
			1214(IR) 1215(R)	CH_3 rock._ (DMG^+^)
			1160(IR) 1161(R)	CH_3 rock._ (DMG^+^), COH _bend._ (CLA^−^)
1177	1175		1154(IR) 1152(R)	CH_3 rock._ (DMG^+^), COH _bend._ (CLA^−^)
1140	1142		1118(IR) 1117(R)	CH_3 rock._ (DMG^+^)
1114	1121		1075(IR) 1071(R)	OH _bend._, CH_3 rock._ (DMG^+^)
			1062(IR) 1057(R)	OH _bend._, CH_3 rock._ (DMG^+^)
1054	1055		1025(IR) 1025(R)	NH^+^ _rock._, CH_3 rock._ (DMG^+^)
1017	1020		990(IR) 989(R)	C−N _str._, C−C _str._, CH_3 rock._ (DMG^+^)
			976(IR) 976(R)	C−N _str._, CH_3 rock._, CH_2 rock._ (DMG^+^)
1001	1002		973(IR) 974(R)	C−N _str._, CH_3 rock._, CH_2 rock._ (DMG^+^)
			962(IR) 961(R)	C−C _str._, COH _bend._ (CLA^−^)
980	983		958(IR) 958(R)	C−C _str._, COH _bend._ (CLA^−^)
957	961		937(IR) 936(R)	C−N _str._, CH_3 rock._, CH_2 rock._ (DMG^+^)
904 886	910 889		891(IR) 888(R)	C−C _str._, C−N _str._ (DMG^+^), OH _tors._ (CLA^−^)
			870(IR) 868(R)	OH _tors._ (CLA^−^)
862	863		859(IR) 856(R)	OH _tors._ (CLA^−^)
839	837		844(IR) 844(R)	C−N _str._ (DMG^+^)
771	790	798 781	817(IR) 816(R)	C−C _str._, C−Cl _str._ (CLA^−^)
747	752	755	762(IR) 762(R)	Ring def. (CLA^−^)
675	677	679	663(IR) 663(R)	COOH _bend._ (DMG^+^)
616	608	621	567(IR) 561(R)	Ring def. (CLA^−^), COOH _rock._ (DMG^+^)
571	573	570	555(IR) 556(R)	CH_2 rock._, COOH _rock._ (DMG^+^)
535	536	539	530(IR) 530(R)	Ring def. (CLA^−^)
504	505	509	504(IR) 505(R)	Ring def. (CLA^−^)
461	466	469	486(IR) 487(R)	C−C−N _bend._, C−N−C _bend._, N−CH_3 bend._ (DMG^+^)
434	436		431(IR) 430(R)	Ring def. (CLA^−^), C−C−N _bend._, N−CH_3 bend._ (DMG^+^)
405	408	417	401(IR) 400(R)	Ring def. (CLA^−^)
			361(IR) 361(R)	Ring def. (CLA^−^), C−C−N _bend._ (DMG^+^)
372	369 352	380 364	358(IR) 356(R)	C=O _bend._, C−O _bend._ (CLA^−^), CH_2 rock._ (DMG^+^)
			340(IR) 340(R)	C−N−C _bend._, N−CH_3 bend._ (DMG^+^), COH _bend._ (CLA^−^)
307			300(IR) 300(R)	Ring def. (CLA^−^)
286	287	284	281(IR) 280(R)	COH _bend._ (CLA^−^), C−N−C _bend._, CH_3 tors._ (DMG^+^)
			266(IR) 266(R)	CH_3 tors._, CH_2 rock._ (DMG^+^)
			232(IR) 232(R)	CH_3 tors._, CH_2 rock._ (DMG^+^)
236	236	235	227(IR) 229(R)	CH_3 tors._ (DMG^+^)
			213(IR) 212(R)	CH_3 tors._ (DMG^+^)
			209(IR) 208(R)	CH_3 tors._ (DMG^+^)
220	214	223	205(IR) 203(R)	Ring tors. C=O _wagg._ (CLA^−^)
208		212	200(IR) 199(R)	Ring tors. C=O _wagg._ (CLA^−^)
		175	178(IR) 178(R)	C−C _tors._ (DMG^+^)
156	163	163	168(IR) 168(R)	C−C _tors._ (DMG^+^)
	141	152	149(IR) 151(R)	
137	135	146	138(IR) 140(R)	Ring tors. (CLA^−^), C−N _tors._ (DMG^+^)
122		127 120	25–135	Lattice vibrations
101 92	92	94		
65		79		
54	59	64		

**Table 7:** The fitted parameters: a, b, c, and *E_a_* for the temperature dependence of FWHM of the infrared band at 434 cm^−1^, associated with the N–CH_3_ bending mode obtained in the temperature range of 290–10 K for the N,N-dimethylglycine–chloranilic acid (DMG^+^–CLA^−^) co-crystal (cm^−1^).

Parameter	Value
a (cm^−1^)	6.53 ± 0.07
b (cm^−1^·K)	2.65 × 10^−3^ ± 8.6 × 10^−5^
c (cm^−1^)	44.00 ± 9.18
*E_a_* (kJ·mol^−1^)	6.7 ± 0.6

## Data Availability

The data presented in this study are available on reasonable request from the corresponding author.

## References

[1] Lane H.-Y., Huang C.-L., Wu P.-L., Liu Y.-C., Chang Y.-C., Lin P.-Y., Chen P.-W., Tsai G. (2006). Glycine Transporter I Inhibitor, N-Methylglycine (Sarcosine), Added to Clozapine for the Treatment of Schizophrenia. Biol. Psychiatry.

[2] Anioła M., Dega-Szafran Z., Katrusiak A., Szafran M. (2014). NH⋯O and OH⋯O Interactions of Glycine Derivatives with Squaric Acid. New J. Chem..

[3] Hildre A.S., Solvang S.-E.H., Aarsland D., Midtun Ø., McCann A., Ervik A.O., Nygård O., Ueland P.M., Nordrehaug J.E., Giil L.M. (2020). Components of the Choline Oxidation Pathway Modify the Association between the Apolipoprotein Ε4 Gene Variant and Cognitive Decline in Patients with Dementia. Brain Res..

[4] Graber C.D., Goust J.M., Glassman A.D., Kendall R., Loadholt C.B. (1981). Immunomodulating Properties of Dimethylglycine in Humans. J. Infect Dis..

[5] Cools A., Maes D., Buyse J., Kalmar I.D., Vandermeiren J.-A., Janssens G.P.J. (2010). Effect of N,N-Dimethylglycine Supplementation in Parturition Feed for Sows on Metabolism, Nutrient Digestibility and Reproductive Performance. Animal.

[6] Gillies N.A., Milan A.M., Chia P.H.P., Sharma P., Mitchell S.M., Zeng N., Ramzan F., D’Souza R.F., Mitchell C.J., Knowles S.O. (2021). Responsiveness of One-Carbon Metabolites to a High-Protein Diet in Older Men: Results from a 10-Wk Randomized Controlled Trial. Nutrition.

[7] Petrosyan A.M., Ghazaryan V.V., Giester G., Fleck M., Tylczyński Z., Wiesner M. (2018). Halogenides of Dimethylglycine in Comparison with Respective Salts of Glycine, Sarcosine and Betaine. J. Mol. Struct..

[8] Fleck M., Petrosyan A.M. (2014). Salts of Amino Acids.

[9] Schaack G. (1990). Experimental Results on Phase Transitions in Betaine Compounds. Ferroelectrics.

[10] Abdinejad T., Zamanloo M.R., Esrafili M.D., Seifzadeh D. (2020). Constructing a Dual-Mode Photochromic and Intrinsically Electrochromic Device Based on Organic Salts Prepared by Acid-Base Neutralization of Pyromellitic Diimides Bearing a Carboxyl Group with Aliphatic Amines. J. Photochem. Photobiol. A Chem..

[11] Balashova E.V., Krichevtsov B.B., Zaitseva N.V., Yurko E.I., Svinarev F.B. (2015). Ferroelectric Thin Films of Deuterated Betaine Arsenate. Ferroelectrics.

[12] Clarke H.T., Gillespie H.B., Weisshaus S.Z. (1933). The Action of Formaldehyde on Amines and Amino Acids1. J. Am. Chem. Soc..

[13] Rodrigues V.H., Paixão J.A., Costa M.M., Beja A.M. (2001). Conformation of Cationic N,N-Dimethylglycine in Dimethylglycinium Trifluoroacetate. Acta Crystallogr. C.

[14] Pawlukojć A., Hetmańczyk J., Nowicka-Scheibe J., Maurin J.K., Schilf W., Rozwadowski Z. (2017). Spectroscopic, Thermal and Structural Studies of New l-Leucine and d-Leucine Complexes with Chloranilic Acid. J. Mol. Struct..

[15] Hetmańczyk J., Nowicka-Scheibe J., Maurin J.K., Pawlukojć A. (2018). Low Temperature Investigations of Dynamic Properties in L-Leucine—Chloranilic Acid Complex. Spectrochim. Acta A Mol. Biomol. Spectrosc..

[16] Zaman M.B., Tomura M., Yamashita Y. (2001). Crystal Engineering Using Anilic Acids and Dipyridyl Compounds through a New Supramolecular Synthon. J. Org. Chem..

[17] Klinman J.P., Mu D. (1994). Quinoenzymes in Biology. Annu. Rev. Biochem..

[18] Gómez-Zavaglia A., Fausto R. (2003). Low-Temperature Solid-State FTIR Study of Glycine, Sarcosine and N,N-Dimethylglycine: Observation of Neutral Forms of Simple α-Amino Acids in the Solid State. Phys. Chem. Chem. Phys..

[19] Matei A., Drichko N., Gompf B., Dressel M. (2005). Far-Infrared Spectra of Amino Acids. Chem. Phys..

[20] Wolpert M., Hellwig P. (2006). Infrared Spectra and Molar Absorption Coefficients of the 20 Alpha Amino Acids in Aqueous Solutions in the Spectral Range from 1800 to 500cm^−1^. Spectrochim. Acta A Mol. Biomol. Spectrosc..

[21] Culka A., Jehlička J., Edwards H.G.M. (2010). Acquisition of Raman Spectra of Amino Acids Using Portable Instruments: Outdoor Measurements and Comparison. Spectrochim. Acta A Mol. Biomol. Spectrosc..

[22] Filho P.F.F., Jiao X., Freire P.T., Lima J.A., dos Santos A.O., Henry P.F., Yokaichiya F., Kremner E., Bordallo H.N. (2011). Structure–Property Relations in Crystalline L-Leucine Obtained from Calorimetry, X-rays, Neutron and Raman Scattering. Phys. Chem. Chem. Phys..

[23] Pawlukojć A., Bator G., Sobczyk L., Grech E., Nowicka-Scheibe J. (2003). Inelastic Neutron Scattering, Raman, Infrared and DFT Theoretical Studies on Chloranilic Acid. J. Phys. Org. Chem..

[24] CrysAlisPro Software System, Agilent Technologies, Version 1.171.33.66; CrysAlis171. NET, Release 28-04-2010. Empirical Absorption Correction Using Spherical Harmonics, Implemented in SCALE3 ABSPACK Scaling Algorithm. https://www.rigakuxrayforum.com/.

[25] Sheldrick G.M. (2008). A Short History of SHELX. Acta Crystallogr. A.

[26] Natkaniec I., Chudoba D., Hetmańczyk L., Kazimirov V.Y., Krawczyk J., Sashin I.L., Zalewski S. (2014). Parameters of the NERA Spectrometer for Cold and Thermal Moderators of the IBR-2 Pulsed Reactor. J. Phys. Conf. Ser..

[27] Parker S.F., Lennon D., Albers P.W. (2011). Vibrational Spectroscopy with Neutrons: A Review of New Directions. Appl. Spectrosc..

[28] Mitchell P.C.H., Parker S.F., Ramirez-Cuesta A.J., Tomkinson J. (2005). Vibrational Spectroscopy with Neutrons: With Applications in Chemistry, Biology, Materials Science and Catalysis.

[29] Pawlukojć A., Sobczyk L. (2004). Application of Inelastic Neutron Scattering (INS) in Studies on Low Frequency Molecular Vibrations. Trends Appl. Spectrosc..

[30] Hohenberg P., Kohn W. (1964). Inhomogeneous Electron Gas. Phys. Rev..

[31] Kohn W., Sham L.J. (1965). Self-Consistent Equations Including Exchange and Correlation Effects. Phys. Rev..

[32] Clark S.J., Segall M.D., Pickard C.J., Hasnip P.J., Probert M.I.J., Refson K., Payne M.C. (2005). First Principles Methods Using CASTEP. Z. Kristallogr. Cryst. Mater..

[33] Pfrommer B.G., Côté M., Louie S.G., Cohen M.L. (1997). Relaxation of Crystals with the Quasi-Newton Method. J. Comput. Phys..

[34] Refson K., Tulip P.R., Clark S.J. (2006). Variational Density-Functional Perturbation Theory for Dielectrics and Lattice Dynamics. Phys. Rev. B.

[35] Perdew J.P., Burke K., Ernzerhof M. (1996). Generalized Gradient Approximation Made Simple. Phys. Rev. Lett..

[36] Tkatchenko A., Scheffler M. (2009). Accurate Molecular Van Der Waals Interactions from Ground-State Electron Density and Free-Atom Reference Data. Phys. Rev. Lett..

[37] Monkhorst H.J., Pack J.D. (1976). Special points for Brillouin-zone integrations. Phys. Rev. B.

[38] Schatschneider B., Monaco S., Tkatchenko A., Liang J.-J. (2013). Understanding the Structure and Electronic Properties of Molecular Crystals Under Pressure: Application of Dispersion Corrected DFT to Oligoacenes. J. Phys. Chem. A.

[39] Dymkowski K., Parker S.F., Fernandez-Alonso F., Mukhopadhyay S. (2018). AbINS: The Modern Software for INS Interpretation. Phys. B Condens. Matter.

[40] Arnold O., Bilheux J.C., Borreguero J.M., Buts A., Campbell S.I., Chapon L., Doucet M., Draper N., Ferraz Leal R., Gigg M.A. (2014). Mantid—Data Analysis and Visualization Package for Neutron Scattering and μ SR Experiments. Nucl. Instrum. Methods A.

[41] Turner M.J., McKinnon J.J., Wolff S.K., Grimwood D.J., Spackman P.R., Jayatilaka D., Spackman M.A. (2017). CrystalExplorer17. University of Western Australia. https://hirshfeldsurface.net.

[42] Pawlukojć A., Hetmańczyk J., Hetmańczyk Ł., Nowicka-Scheibe J., Maurin J.K., Schilf W., Trzybiński D., Woźniak K. (2021). Evidence of Low Temperature Phase Transition in 2,6-Dimethylpyrazine—Picric Acid Cocrystal by Means of Temperature Dependent Investigations: X-Ray, DSC and IR. J. Mol. Struct..

[43] Pawlukojć A., Sobczyk L., Prager M., Bator G., Grech E., Nowicka-Scheibe J. (2008). DFT Calculations of 2,6-Dimethylpyrazine (26DMP) and Its Complex with Chloranilic Acid (CLA): Comparison to INS, IR and Raman Vibration Spectra. J. Mol. Struct..

[44] Jmol: An Open-Source Java Viewer for Chemical Structures in 3D. http://www.jmol.org/.

[45] Sawka-Dobrowolska W., Bator G., Sobczyk L., Grech E., Nowicka-Scheibe J., Pawlukojć A. (2005). Structure and Vibrational Spectra of 1:1 Chloranilic Acid (CLA)—Tetramethylpyrazine (TMP) Complex. Struct. Chem..

[46] Bator G., Sawka-Dobrowolska W., Sobczyk L., Owczarek M., Pawlukojć A., Grech E., Nowicka-Scheibe J. (2012). Hydrogen Bonded NHO Chains Formed by Chloranilic Acid (CLA) with 4,4′-Di-t-Butyl-2,2′-Bipyridyl (DtBBP) in the Solid State. Chem. Phys..

[47] Carabatos-Nédelec C., Becker P. (1997). Order–Disorder and Structural Phase Transitions in Solid-State Materials by Raman Scattering Analysis. J. Raman Spectrosc..

[48] da R. Andrade P., Rao A.D.P., Katiyar R.S., Porto S.P.S. (1973). Analysis of the Relationship between Temperature Dependence of the Libration Mode and Dielectric Relaxation in NaNO_2_. Solid State Commun..

[49] da R. Andrade P., Porto S.P.S. (1973). On Linewidth of Phonons Associated to a Disorder Mechanism. Solid State Commun..

